# Comparative Phytochemical Characterization and Bioactivity Profiling of Serbian *Ajuga* Species from Different Localities: Antioxidant, Antityrosinase, Antimicrobial and Anti-Pyocyanin Activities

**DOI:** 10.3390/plants15152306

**Published:** 2026-07-27

**Authors:** Ana M. Džamić, Jelena S. Matejić, Ksenija S. Mileski, Lazar D. Žarković, Marija S. Marković, Anđela V. Dragićević, Uroš M. Gašić, Danijela M. Mišić, Ana D. Ćirić

**Affiliations:** 1 Institute of Botany and Botanical Garden “Jevremovac”, Faculty of Biology, University of Belgrade, Studentski trg 16, 11000 Belgrade, Serbia; ksenija.mileski@bio.bg.ac.rs (K.S.M.); lazar.zarkovic@bio.bg.ac.rs (L.D.Ž.); 2 Faculty of Medicine, University of Niš, Bulevar Dr Zorana Đinđića 81, 18000 Niš, Serbia; dragicevic.andjela@gmail.com; 3 Institute of Forestry—National Institute of the Republic of Serbia, University of Belgrade, Kneza Višeslava 3, 11030 Belgrade, Serbia; markovicsmarija9@gmail.com; 4 Institute for Biological Research “Siniša Stanković”—National Institute of the Republic of Serbia, University of Belgrade, Bulevar Despota Stefana 142, 11000 Belgrade, Serbia; urosgasic@gmail.com (U.M.G.); dmisic@ibiss.bg.ac.rs (D.M.M.); rancic@ibiss.bg.ac.rs (A.D.Ć.)

**Keywords:** antimicrobials, antioxidants, BSA, phenolics, tyrosinase, UHPLC

## Abstract

Herbaceous and subshrub species of the genus *Ajuga* are recognized as valuable sources of bioactive metabolites with diverse pharmacological properties. This study comparatively evaluated the phytochemical composition and the in vitro biological activities of *Ajuga chamaepitys*, *A. genevensis*, *A. laxmannii*, and *A. reptans* collected from different localities in Serbia. UHPLC-MS/MS profiling identified *p*-hydroxybenzoic acid and ferulic acid as the predominant phenolic compounds. *A. reptans* exhibited the highest total phenolic content (120.52 mg GAE/g dw), along with the strongest antioxidant activity in DPPH, ABTS, β-carotene bleaching, and FRAP assays, as well as the highest antibacterial activity. *A. laxmannii* contained the highest flavonoid content (48.95 mg QuE/g dw) and showed the strongest antifungal activity, whereas *A. genevensis* demonstrated the most potent tyrosinase inhibitory activity. Notably, *A. genevensis* extracts exhibited pronounced anti-pyocyanin activity, approximately twofold stronger than ampicillin. These findings demonstrate significant species- and locality-dependent variation in the phytochemical composition and biological activity and identify Serbian *Ajuga* species as promising natural sources of antioxidant, antimicrobial, and antivirulence compounds with potential pharmaceutical and food applications.

## 1. Introduction

The genus *Ajuga* L. (Lamiaceae) comprises flowering plants widely distributed in temperate regions, including Asia, Europe, Australia, North America, and Africa [1]. It is estimated that the genus comprises more than 300 species, including about 135 subspecies and varieties [2,3]. Ten *Ajuga* species are recognized in the Flora Europeaea [4], of which five have been recorded in the Flora of Serbia [5]. These species are annual and perennial herbaceous plants characterized by rapid growth and a ground-covering habit. Some varieties have attractive leaves and flowers, making them popular ornamental plants in horticulture, while also possessing ecological, medicinal, and economic value [2]. The most biologically active compounds identified in *Ajuga* species, with considerable therapeutic potential, are phytoecdysteroids, sesquiterpenoids, clerodane diterpenes, triterpenoid steroids, essential oils, iridoid glycosides, and withanolides [3,6,7]. In traditional medicine, these plants have been used to treat inflammation, diabetes mellitus, fever, malaria, pneumonia, tuberculosis, rheumatism, gastrointestinal disorders, hypertension, and pain [2,8]. Their extracts exhibit antioxidative, anti-inflammatory, antimicrobial, cytotoxic, antiproliferative, antimutagenic, and neuroprotective activities [9,10,11,12]. *Ajuga* species are widely used in Turkish folk medicine for their purported health effects and homeopathic applications [13]. An ethnobotanical study on the traditional uses of wild medicinal plants in the Prokletije Mountains (Montenegro) recorded the use of aerial parts of *A. reptans* for the treatment of gallbladder and stomach disorders and, when applied externally, for inflammation of the mouth and larynx [14]. Łuczaj et al. (2021) [15] reported that *A. chamaepitys* was used on the Adriatic Islands as a medicinal infusion, an ingredient in medicinal rakija, and a panacea. In the mountainous region of the Zavidovići municipality (BiH), natural habitats of many medicinal plants, including blue bugle (*Ajuga reptans* L.), have been documented. However, the traditional use of this species was not mentioned by the authors [16].

According to the literature, the studied *Ajuga* species (*Ajuga chamaepitys*, *A. genevensis*, *A. laxmannii,* and *A. reptans*) are characterized as follows:

*Ajuga genevensis* L. (blue bugle, blue bugleweed, Geneva bugleweed) is a pubescent plant native to Europe, typically occurring in dry, well-lit habitats. High contents of rosmarinic, oleanolic, and maslinic acids have been reported in *A. genevensis* collected in Italy [17]. According to Venditti et al. (2016) [17], extracts of *A. genevensis* also possess strong antioxidant, neuroprotective, and antiproliferative activities.

*A. chamaepitys* (L.) Schreb. (yellow bugle, ground-pine), a typical West European plant, grows throughout the region at elevations of up to 1600 m on warm slopes. It is native to central and southern Europe, including the eastern Mediterranean region, and North Africa, whereas no species are native to the rest of the Western Hemisphere [18]. Previous studies have shown that its bioactive compounds include flavanones such as isovitexin and orientin, the flavonol chrysoeriol 7-*O*-glucopyranoside, the flavone glycoside apigenin 7-*O*-rhamnopyranoside [19], and iridoids such as ajugoside, asperulosidic acid, reptoside, 8-*O*-acetylharpagide, harpagide, 5-*O*-*β*-D-glucopyranosyl-harpagide, deacetylasperulosidic acid, and 5-*O*-*β*-D-glucopyranosyl-8-*O*-acetylharpagide [17]. Isovitexin and orientin were reported by [20]. Infusions prepared from the aerial parts of the plant are used to treat hemorrhoids, diarrhea, and various intestinal disorders. The plant has also been reported to possess insecticidal properties [21]. Furthermore, its essential oil exhibited moderate cytotoxic activity against the tested cell lines (A375 human malignant melanoma, MDA-MB-231 human breast adenocarcinoma, and HCT116 human colon carcinoma), as determined by the methyl thiazole tetrazolium (MTT) assay [17].

*A. laxmannii* (Murray) Benth. (bugleweed, ground-pine) is found in Central and Southeast Europe, Siberia, the Caucasus, and the dry regions of Asia Minor. Toiu et al. (2018) [22] identified caffeic acid derivatives (including chlorogenic acid), flavonoid glycosides (isoquercitrin, rutin, and quercitrin), free aglycones (luteolin and apigenin), iridoids (including 8-*O*-acetylharpagide), and phytosterols (including *β*-sitosterol) as the major constituents of extracts prepared from the aerial parts of *A. laxmannii*. The presence of polyphenolic compounds, iridoids, and phytosterols in these extracts has been associated with antioxidant, antimicrobial, and anti-inflammatory effects [22]. Extracts from the aerial parts of this species have shown anticancer potential against B16.F10 murine melanoma and C26 colon carcinoma cells [18]. There is evidence that the aerial parts of this plant have been traditionally used to treat respiratory infections [23] and as an adjunct in cancer therapy [24]. In folk medicine, the plant is also used as a galactagogue and for the treatment of inflammation.

*A. reptans* L. (bugle, common bugle, or bugleweed) is widespread in Europe, prefers wetter habitats, and is characterized by rapid vegetative spread. This plant has traditionally been used for the treatment of liver disorders, diabetes, diarrhea, ulcers, hypertension, urinary tract and cardiovascular disorders, as well as to delay premature cell ageing [25]. According to published studies, extracts obtained from *A. reptans* are rich in flavonoids, iridoids, triterpenes, diterpenes, ecdysteroids, and sterols, which contribute to their antioxidant, antimicrobial, and anti-inflammatory activities [8,26,27]. Göger et al. (2021) [28] reported strong antioxidant and antimicrobial activities in extracts prepared from the aerial parts of *A. reptans*. Moreover, cytotoxic evaluation of aerial part extracts (flowers and leaves analyzed separately) using the MTT assay demonstrated antiproliferative activity against prostate and lung cancer cells [29].

Characterization of the biological activities of medicinal plants can facilitate the identification of novel therapeutic compounds. The rapid spread of antimicrobial resistance and the limited long-term effectiveness of newly developed antibiotics have intensified the search for plant-derived alternatives. According to the World Health Organization (WHO), medicinal plants are important sources of therapeutic agents [30], while extracts that inhibit pathogenic microorganisms and exhibit low toxicity toward host cells are promising candidates for the development of novel antimicrobial agents [31].

Although the phytochemical composition and the conventional biological activities of *Ajuga* species have been extensively investigated, comparative studies of geographically diverse Serbian populations remain limited. Moreover, little attention has been paid to their antivirulence potential, particularly their ability to inhibit pyocyanin production in *Pseudomonas aeruginosa*, which represents an emerging strategy for reducing bacterial pathogenicity without directly affecting bacterial growth [32].

Therefore, this study comparatively evaluated the phytochemical composition and biological activities of extracts obtained from the aerial parts of four *Ajuga* species (*A. laxmannii* (Murray) Benth., *A. chamaepitys* (L.) Schreb., *A. reptans* L., and *A. genevensis* L.) collected from different localities in Serbia. The main objectives were to: (i) identify and quantify phenolic compounds; (ii) evaluate antioxidant activity; (iii) determine antibacterial, antifungal, and anti-pyocyanin activities; (iv) assess anti-inflammatory and tyrosinase-inhibitory activities; and (v) examine the relationships between phenolic composition and the investigated biological activities through correlation analysis. Particular emphasis was placed on the evaluation of anti-pyocyanin activity as an emerging antivirulence strategy against *Pseudomonas aeruginosa*, while the selected biological assays were designed to provide a comprehensive evaluation of the pharmacological potential of Serbian *Ajuga* species and to contribute to the scientific validation of their traditional use.

## 2. Results and Discussion

### 2.1. Chemical Composition

#### 2.1.1. Ultra-High-Performance Liquid Chromatography-Tandem Mass Spectrometry (UHPLC-MS/MS) Analysis of Phenolic Compounds

The phenolic composition of *Ajuga* extracts was analyzed by UHPLC-MS/MS, and the results are presented in Table 1. In total, 19 polyphenolic compounds were identified and quantified in all analyzed extracts. Eight free phenolic acids were detected, including three hydroxybenzoic acids (protocatechuic acid, *p*-hydroxybenzoic acid, and gentisic acid) and five hydroxycinnamic acids (caffeic acid, *p*-coumaric acid, 5-*O*-caffeoylquinic acid, ferulic acid, and rosmarinic acid) (Table 1). Additionally, four flavones and one flavone glycoside (luteolin, apigenin, hispidulin, cirsimaritin, and vitexin), two flavanones and one flavanone glycoside (eriodictyol, naringenin, and naringin), as well as one flavonol and two flavonol glycosides (kaempferide, rutin, and quercetin 3-*O*-glucoside) were identified in the samples. The most abundant compounds were *p*-hydroxybenzoic acid and ferulic acid, which were present in most *Ajuga* samples at concentrations above 1 mg/kg dw (Table 1).

The highest concentrations were found in sample A5 for *p*-hydroxybenzoic acid (1.8 mg/kg dw) and in sample A2 for ferulic acid (5.81 mg/kg dw) (Table 1). The flavonol kaempferide was detected only in three of the nine analyzed samples (A3, A6, and A7) at levels below 0.02 mg/kg dw. By contrast, the flavone cirsimaritin was present in five samples (A1, A2, A6, A7, and A8) at concentrations below 0.08 mg/kg dw. In the extract prepared from the aerial part of *A. laxmannii* (A1), the predominant detectable compound was the flavone glycoside vitexin (1.51 mg/kg dw), followed by ferulic acid (1.16 mg/kg dw) and *p*-hydroxybenzoic acid (0.72 mg/kg dw). In the extract prepared from the aerial parts of *A. chamaepitys* (A2), ferulic acid was the major compound (5.81 mg/kg dw), followed by *p*-hydroxybenzoic acid (1.78 mg/kg dw). Additionally, the flavone luteolin was present at a significant concentration (1.55 mg/kg dw) in sample A2. The extracts of *A. reptans* (A3, A4, A5, and A6) demonstrate how local environmental conditions can influence phytochemical composition (Table 1). In extracts A3 and A4, the flavanone glycoside naringin was detected at concentrations of 2.54 mg/kg dw and 1.51 mg/kg dw, respectively. The predominant phenolic acids were the hydroxycinnamic acid 5-*O*-caffeoylquinic acid (2.48 mg/kg dw) in A3 and *p*-hydroxybenzoic acid in A4, respectively (Table 1). Interestingly, no *p*-hydroxybenzoic acid was detected in extract A3, whereas no 5-*O*-caffeoylquinic acid was detected in extract A4, and no gentisic acid was detected in extract A6 (Table 1). Rutin was detected at its lowest concentration in A4 (0.01 mg/kg dw). Among the flavanones, naringenin was consistently present at a low concentration (0.01 mg/kg dw) in all *A. reptans* extracts (Table 1). As with the extracts of *A. laxmannii* and *A. chamaepitys*, ferulic acid was the predominant compound in *A. genevensis* extracts, with concentrations of 2.0 mg/kg dw in A7 and 2.14 mg/kg dw in A9 (Table 1). Furthermore, as observed in the *A. reptans* extract A5, *p*-hydroxybenzoic acid was the predominant compound in *A. genevensis* extract A8, with a concentration of 1.31 mg/kg dw. Naringenin was consistently detected at the lowest concentration among the identified flavonoids in both *A. genevensis* extracts (A8 and A9), as was the case for the *A. reptans* extracts.

In the *A. laxmannii* extract, rutin was detected at a low concentration (0.025 mg/kg dw), second only to naringenin. However, previous studies have reported that rutin is the primary compound present at relatively high concentrations [18,22]. Isoquercitrin, a flavonoid glycoside with potent anti-inflammatory properties, was also identified and quantified in ethanol and methanol extracts of *A. laxmannii* [22]. Additionally, HPLC-MS analysis of *A. laxmannii* aerial parts revealed the presence of iridoid glycosides, specifically aucubin, catalpol, harpagide, harpagoside, and 8-*O*-acetylharpagide [22]. According to [18], luteolin was the most abundant compound in the ethanol extract of *A. chamaepitys*, which is consistent with our results, in which luteolin was the second most abundant compound. In the dry extract obtained from the aerial parts of *A. chamaepitys* using 50% (*v*/*v*) ethanol, caffeic acid, kaempferol, and genistin were the most abundant compounds, with the concentrations of 3253.8, 3041.5, and 730.2 μg/g dry extract, respectively, as determined by UHPLC-HRMS/MS [21]. Isoquercitrin, also known as quercetin-3-glucoside, was reported as the major polyphenol in ethanol and methanol extracts of *A. reptans* (180.77 ± 2.84 and 151.10 ± 2.77 µg/g dw, respectively), while luteolin was the predominant compound in ethanol and methanol extracts of *A. genevensis* (46.16 ± 1.93 and 42.97 ± 1.89 µg/g dw, respectively) [8]. Notably, the flavonoid glycosides isoquercitrin and rutin were identified only in *A. reptans* extracts, whereas the phenolic acids caffeic acid and *p*-coumaric acid were detected only in *A. genevensis* extracts [8].

The predominance of ferulic acid and *p*-hydroxybenzoic acid observed in the investigated *Ajuga* species is consistent with reports on several medicinal plants rich in phenolic acids, including *Salvia officinalis*, *Rosmarinus officinalis*, *Melissa officinalis* and *Origanum vulgare*, in which these compounds contribute substantially to antioxidant, antimicrobial, and anti-inflammatory activities [33,34]. Similar biological effects have also been attributed to ferulic acid in medicinal species of the Lamiaceae and Asteraceae families, supporting the conclusion that the activities observed in the present study are in agreement with the known pharmacological properties of these phenolic constituents [35].

To better understand the phytochemical diversity and screen for chemotaxonomic markers among the investigated *Ajuga* extracts, a Principal Component Analysis (PCA) was performed based on the quantified phenolic compounds (Figure S1). The first two principal components accounted for 53.24% of the total variance (PC1: 28.79%, PC2: 24.46%), demonstrating a clear spatial separation driven by both species characterization and geographical origin. The extracts obtained from *A. reptans* L. (A3, A4, A5, and A6) clustered distinctly on the right side of the biplot along the positive PC1 axis. This segregation was strongly driven by their higher concentrations of hydroxycinnamic acids, most notably caffeic acid and 5-O-caffeoylquinic acid. *A. genevensis* differentiation: Conversely, *A. genevensis* L. samples (A7, A8, and A9) clustered towards the lower-left quadrant, showing a distinct chemical profile characterized by specific flavonoid patterns. Interestingly, *A. laxmannii* (A1) from Vidlič Mountain was separated into the upper-left quadrant, heavily influenced by its unique abundance of vitexin and rosmarinic acid. These results suggest that while genetic factors primarily determine the baseline metabolic profile, the microclimate and soil conditions of the localities (e.g., Vidlič vs. Suva planina) induce notable quantitative shifts in secondary metabolite accumulation.

#### 2.1.2. Total Phenolic (TPC) and Total Flavonoid Content (TFC)

As part of the chemical composition analyses, the results for total phenolic and flavonoid contents in *Ajuga* extracts are summarized in Table 2.

**Figure 1:**
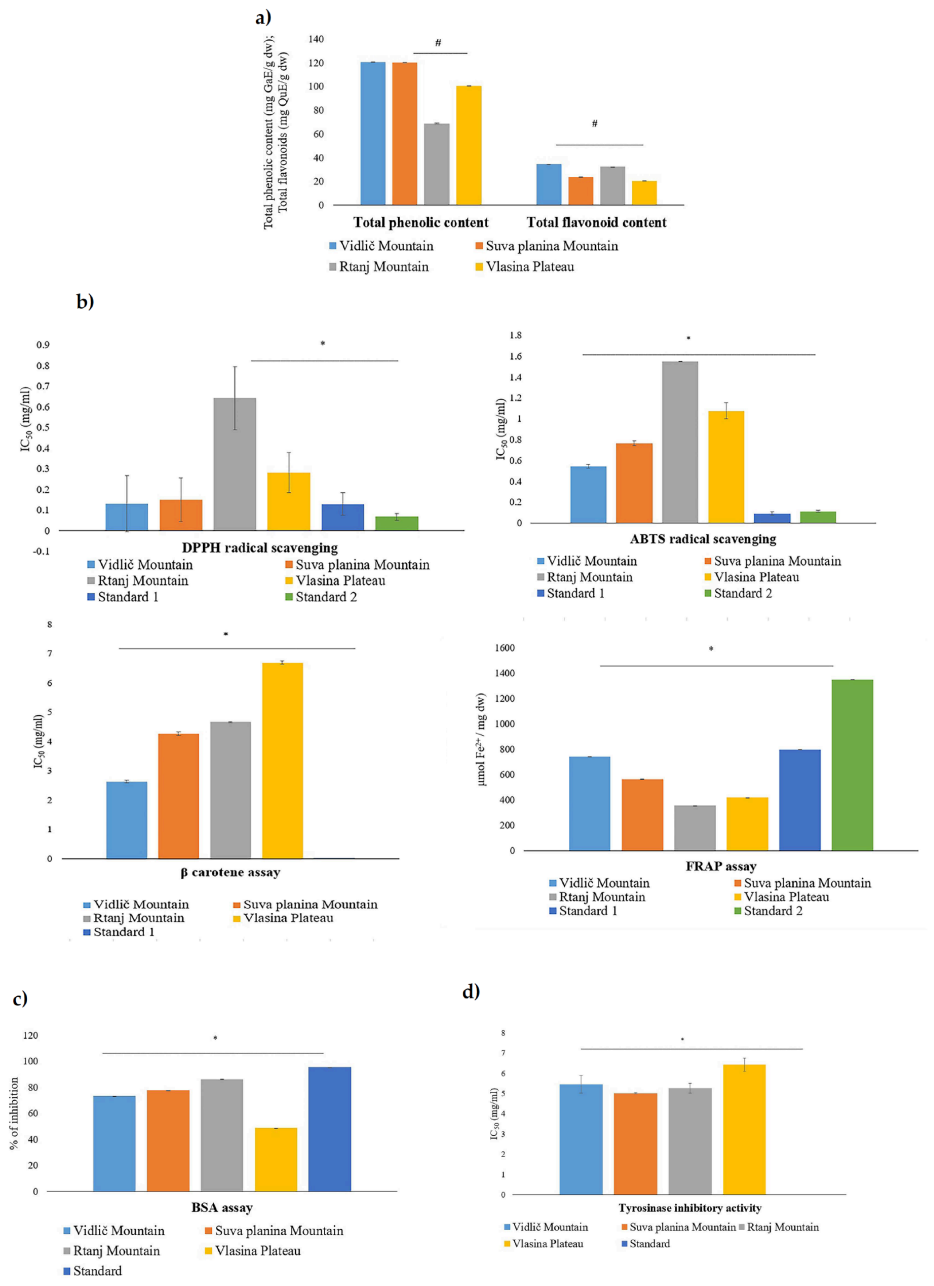
Influence of different localities on the biological activities of *Ajuga reptans* (A3—Vidlič Mountain, A4—Suva Planina Mountain, A5—Rtanj Mountain, A6—Vlasina Plateau): (**a**) total phenolic content (TPC) and total flavonoid content (TFC); (**b**) antioxidant activity (standard 1: BHA, standard 2: vitamin C); (**c**) anti-inflammatory activity (standard: diclofenac); (**d**) tyrosinase inhibitory activity (standard: kojic acid). Data are presented as the mean ± SD and were analyzed using one-way ANOVA followed by Tukey’s post hoc test. # *p* < 0.05 (# indicates differences among samples); * *p* < 0.05 (* indicates differences between the samples and the corresponding standards).

**Figure 2:**
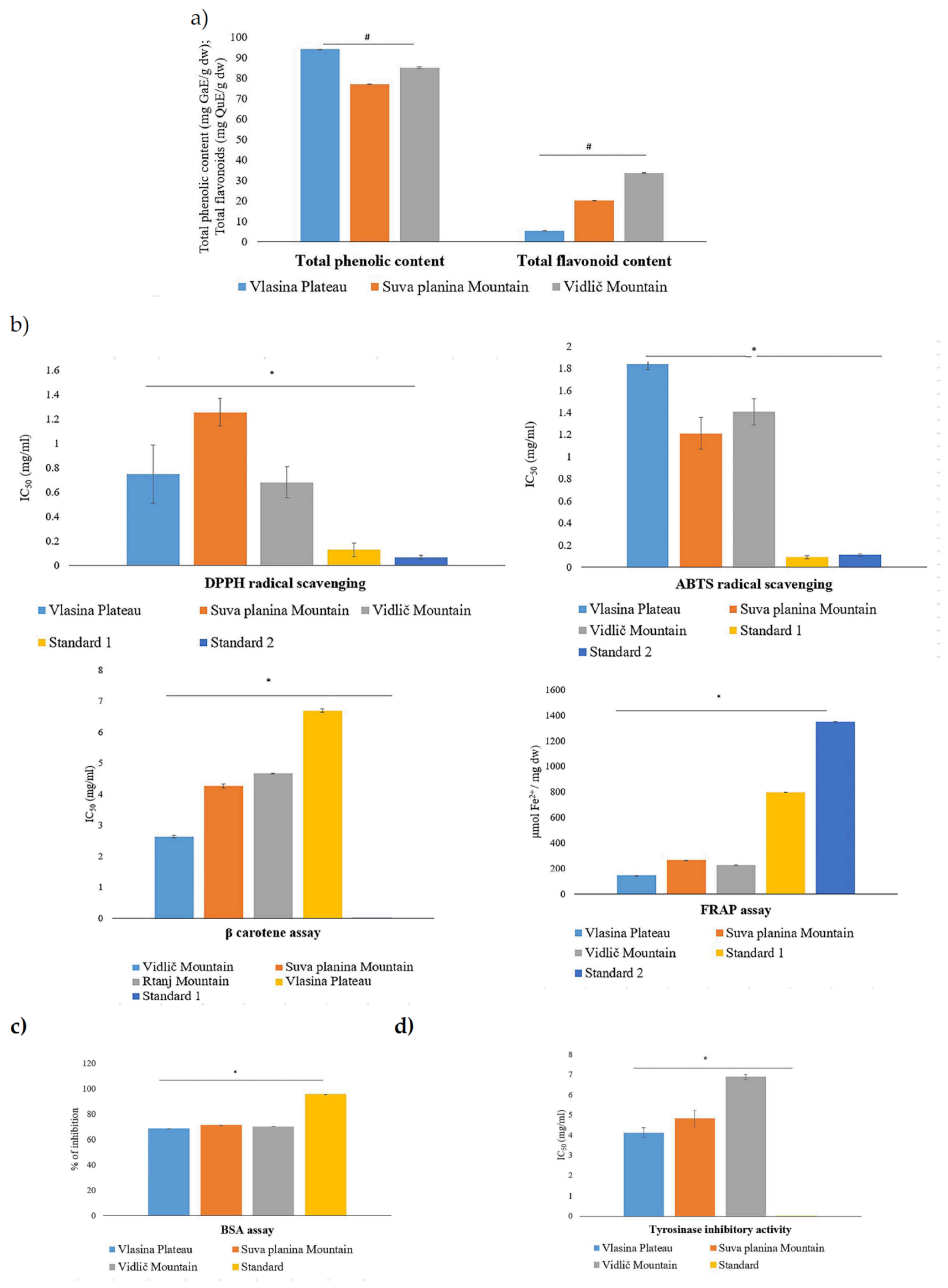
Influence of collection locality on the biological activities of *Ajuga genevensis* extracts (A7—Vlasina Plateau, A8—Suva Planina Mountain, A9—Vidlič Mountain): (**a**) total phenolic content (TPC) and total flavonoid content (TFC); (**b**) antioxidant activity (standard 1: BHA, standard 2: vitamin C); (**c**) anti-inflammatory activity (standard: diclofenac); (**d**) tyrosinase inhibitory activity (standard: kojic acid). Data are presented as the mean ± SD and further compared using one-way ANOVA followed by Tukey’s post hoc test. # *p* < 0.05 (# indicates differences among samples); * *p* < 0.05 (* indicates a difference between the samples and the corresponding standards).

The total phenolic content (TPC) of the investigated *Ajuga* extracts ranged from 68.91 to 120.52 mg GAE/g dw, whereas the total flavonoid content (TFC) ranged between 5.47 and 48.94 mg QuE/g dw. In general, TPC values were higher than TFC values in all analyzed extracts. Among the tested samples, *A. reptans* extracts A3 and A4 exhibited the highest TPC, while *A. laxmannii* (A1) contained the highest TFC. Significant differences (*p* < 0.05) were observed among most samples, indicating that both species identity and collection locality influenced the accumulation of phenolic compounds. As shown in Figure 1a, the TPC and TFC of *A. reptans* varied significantly among collection localities, although samples A3 (Vidlič Mountain) and A4 (Suva Planina Mountain) did not differ significantly. Similarly, Figure 2a shows that the collection locality also affected the TPC and TFC of *A. genevensis*.

The TPC and TFC values obtained for *A. laxmannii* are in good agreement with previous reports. Toiu et al. [22] reported TPC values of 67.68 ± 1.57 and 56.76 ± 0.92 mg GAE/g dw and TFC values of 36.14 ± 0.53 and 31.22 ± 0.39 mg RE/g dw for ethanol and methanol extracts, respectively, which are comparable to the values obtained in the present study. Likewise, Toiu et al. [18] demonstrated that ethanol extracts of *A. laxmannii* contained higher total phenolic and total flavonoid contents than *A. genevensis*, whereas the latter contained higher levels of iridoids, supporting the species-dependent differences observed in our results.

For *A. chamaepitys*, the measured TPC was comparable to previously published values for ethanolic extracts (83.31 ± 3.98 mg TAE/g dw) [21]. However, higher TFC values have been reported for extracts obtained with ethyl acetate [36], confirming that solvent polarity strongly influences the extraction efficiency of phenolic constituents. Because flavonoids differ considerably in polarity, less polar solvents may preferentially extract specific subclasses, resulting in higher TFC values than those obtained with ethanol.

Compared with the study by Toiu et al. [8], our *A. reptans* and *A. genevensis* extracts contained substantially higher amounts of TPCs and TFCs. The higher values observed in the present study may be attributed to differences in geographical origin, environmental conditions, plant developmental stage, and extraction procedures, all of which are known to influence the biosynthesis and accumulation of phenolic metabolites. These findings highlight the importance of both species identity and collection locality in determining the phytochemical composition of *Ajuga* extracts.

### 2.2. Antioxidant Activity

The antioxidant capacity of *Ajuga* extracts was evaluated using four different assays: DPPH, ABTS, *β*-carotene bleaching, and FRAP (Table 2). The highest antioxidant capacity in all assays was observed in sample A3 (*A. reptans*). In the DPPH assay, its activity was comparable to that of the BHA standard, whereas in the FRAP assay, A3 exhibited strong reducing power but remained significantly lower than the BHA standard (Table 2). Sample A7 (*A. genevensis*) exhibited the lowest free radical scavenging activity and reducing power, except in the DPPH assay, where it showed moderate activity. Overall, the standards used were more effective than the tested extracts (Table 2). *Ajuga* extracts exhibited moderate to high free radical scavenging activity. The results of the DPPH assay indicate that samples A3, A4, and A2 were the most potent antioxidants, with activities comparable to that of BHA and only slightly lower than that of vitamin C. Extract A3 exhibited the highest antioxidant capacity (IC_50_ = 0.13 mg/mL), while extract A8 showed the lowest antioxidant activity (IC_50_ = 1.25 mg/mL). The results revealed statistically significant differences (*p* < 0.05) in DPPH free radical scavenging activity between samples A1 and A3, A1 and A4, A5 and A2, A5 and A3, and A5 and A4. Additionally, sample A7 differed significantly (*p* < 0.05) from all other samples, except A1, A5, and A9. Similarly, sample A8 showed statistically significant differences (*p* < 0.05) compared with all other analyzed samples, whereas sample A9 differed significantly from all samples except A1, A5, and A7. Both positive controls (BHA and vitamin C) differed significantly (*p <* 0.05) from all analyzed samples. When evaluating the influence of collection locality on the DPPH free radical scavenging activity of extracts prepared from the aerial parts of *A. reptans*, a statistically significant difference was observed only between the extract from the Rtanj Mountain (A5) and the other samples (*p* < 0.05), as shown in Figure 1b. For *A. genevensis*, no statistically significant difference in DPPH free radical scavenging activity was observed between samples A7 and A9, collected from the Vlasina Plateau and Vidlič Mountain, respectively (Figure 2b). In the ABTS assay, A3 and A4 again exhibited the highest activity, although their activities remained slightly lower than those of the reference standards. Sample A7 showed the lowest ABTS•+ radical scavenging activity. Statistically significant differences (*p* < 0.05) in ABTS•+ radical scavenging activity were observed between most samples and the reference standards, except for the following comparisons: A1 vs. A2, A1 vs. A7, A2 vs. A5, A5 vs. A9, and A6 vs. A8. Collection locality influenced the ABTS•+ radical scavenging activity of extracts prepared from the aerial parts of *A. reptans*. In contrast, no statistically significant difference was observed between *A. genevensis* samples A8 and A9, collected from Suva Planina Mountain and Vidlič Mountain, respectively. In the *β*-carotene bleaching assay, peroxyl (COO•) radicals were scavenged most effectively by A3 and A1, with IC_50_ values of 2.63 ± 0.04 and 2.86 ± 0.01 mg/mL, respectively, whereas extract A7 exhibited the lowest activity (IC_50_ = 8.01 ± 0.01 mg/mL). Statistically significant differences (*p* < 0.05) were found among the IC_50_ values of all samples, except between samples A2 and A4. All samples exhibited significantly lower antioxidant activity than BHA, which had an IC_50_ value of 0.01 mg/mL (*p* < 0.05) (Table 2). In the FRAP assay, *Ajuga* extracts were tested at a concentration of 1 mg/mL. The results further confirmed that sample A3 possessed the highest antioxidant activity, whereas sample A7 exhibited the weakest effect, with FRAP values of 741.81 ± 0.03 and 145.61 ± 0.01 μmol Fe^2+^/mg dw, respectively (Table 2). Sample A3 and the BHA standard exhibited similar FRAP values (741.81 ± 0.03 and 797.05 ± 1.00 μmol Fe^2+^/mg dw), respectively, but both were less effective than vitamin C, which showed a FRAP value of 1348.47 ± 2.00 μmol Fe^2+^/mg dw. Additionally, the FRAP results showed statistically significant differences (*p* < 0.05) among all tested samples and between each sample and the reference standards. Comparative analysis of extracts prepared from the aerial parts of *A. reptans* and *A. genevensis* collected from different localities revealed statistically significant differences (*p* < 0.05) in their ability to prevent *β*-carotene bleaching and in their overall antioxidant capacity, as assessed by the FRAP assay (Figure 1b and Figure 2b). The antioxidant activity observed in the present study is generally consistent with previous reports on *Ajuga* species, although some quantitative differences were evident. The *A. laxmannii* extract (A1) exhibited moderate antioxidant activity in both the DPPH and ABTS assays, which is in agreement with the findings of Toiu et al. [22], who reported DPPH IC_50_ values of 22.64 ± 0.88 and 24.89 ± 0.83 µg/mL for ethanol and methanol extracts, respectively, together with an ABTS radical-scavenging capacity of 71.07 ± 2.40 mg TE/g extract. Similarly, the antioxidant activity of *A. chamaepitys* observed in this study agrees with previous reports showing that ethanolic and acetone extracts possess considerable radical-scavenging capacity, although the activity strongly depends on the extraction solvent [21,36]. The higher antioxidant activity reported for acetone extracts further confirms that solvent polarity plays an important role in the extraction of antioxidant constituents.

Our results for *A. reptans* and *A. genevensis* are also consistent with previous studies in demonstrating that *A. reptans* possesses stronger antioxidant activity than *A. genevensis* [8,25]. In the present study, sample A3 (*A. reptans*, Vidlič Mountain) exhibited the lowest DPPH and ABTS IC_50_ values and the highest FRAP value among all analyzed extracts, whereas *A. genevensis* sample A7 showed the weakest antioxidant activity. Although the absolute IC_50_ values differ from those reported by Toiu et al. [8] and Göger et al. [25], the overall trend is consistent. These discrepancies may be attributed to differences in geographical origin, environmental conditions, extraction procedures, and the phenolic composition of the analyzed samples, all of which are known to influence antioxidant capacity.

Overall, the higher antioxidant activity of the *A. reptans* extracts in the present study is consistent with their higher total phenolic content, supporting the contribution of phenolic compounds to the antioxidant potential of *Ajuga* species.

Hierarchical clustering analysis (HCA) was employed to simultaneously visualize and group the multi-target biological performance of the extracts (Figure S2). The resulting clustered heatmap revealed a prominent functional grouping that correlates tightly with the total phenolic content (TPC) and total flavonoid content (TFC). Samples A3 and A4 (*A. reptans* collected from Vidlič and Suva planina, respectively) formed a distinct cluster characterized by the highest Z-scores for total phenols and FRAP activity. Concurrently, these extracts exhibited the lowest Z-scores for DPPH and ABTS IC_50_ values. Since lower IC_50_ values indicate superior radical scavenging potency, this cluster represents the most effective antioxidant fraction among all tested samples. On the other hand, *A. genevensis* and *A. chamaepitys* extracts displayed moderate to high Z-scores for enzyme inhibition (e.g., Tyrosinase IC_50_), suggesting that their biological mechanism may favor specific enzyme-substrate interactions rather than non-specific electron-transfer pathways. The heatmap successfully validates that the overall antioxidant powerhouse behavior of *A. reptans* is highly synergistic with its rich total phenolic yield.

To mathematically characterize and confirm the kinetic efficiency of the most promising candidates, non-linear regression analysis was applied to construct dose–response curves for the top-performing extracts against DPPH radicals (Figure S3). Sample A3 (*A. reptans*—Vidlič Mountain) emerged as the most potent free radical scavenger, reaching its half-maximal inhibitory concentration at an IC_50_ of 0.13 mg/mL. It was closely followed by sample A4 (*A. reptans*—Suva planina) with an IC_50_ of 0.15 mg/mL, and sample A2 (*A. chamaepitys*) with an IC_50_ of 0.16 mg/mL. All three extracts exhibited a classic, steep sigmoidal transition, achieving near-complete radical scavenging saturation (>90%) within a narrow concentration window (<0.6 mg/mL). The sharp slope of these curves highlights not only the quantity but also the high quality and reactivity of the hydrogen-donating polyphenols present in these specific extracts.

### 2.3. Protein Denaturation

The mechanism underlying protein denaturation is complex and involves alterations in hydrophobic interactions, disulfide bonds, electrostatic interactions, and hydrogen bonds. In inflammatory diseases such as diabetes mellitus, cancer, and rheumatoid arthritis, protein denaturation leads to the formation of autoantigens [37]. Accordingly, inhibition of protein denaturation may help reduce inflammatory activity. The reference drug used in this study was the nonsteroidal anti-inflammatory drug (NSAID) diclofenac. NSAIDs exert their anti-inflammatory effects primarily by inhibiting cyclooxygenase (COX) enzymes. However, these drugs can cause adverse effects such as constipation, ulcers, bleeding, and perforation [38]. The anti-inflammatory activity of the different extracts prepared from the aerial parts of *Ajuga* species was evaluated using the BSA denaturation assay (Table 2). An inhibition of protein denaturation greater than 20% is generally considered indicative of anti-inflammatory activity. As shown in Table 2, the most active extracts in inhibiting BSA denaturation were those of *A. reptans* in the following order: A5 > A4 > A3 (86.23 ± 0.005%, 77.68 ± 0.004%, 73.42 ± 0.005%, respectively), compared with diclofenac (0.1 mg/mL, 95.6% inhibition). The lowest activity among the tested extracts was observed for *A. laxmannii*, with 44.44 ± 0.001% inhibition of BSA denaturation (Table 2). There is a statistically significant difference (*p* < 0.05) in the ability of the tested extracts to inhibit BSA. In addition, a statistically significant difference (*p* < 0.05) was found between the samples of *A. reptans* (A3, A4, A5, and A6) and *A. genevensis* (A7, A8, and A9) collected at different localities (Figure 1c and Figure 2c). Furthermore, a significant difference (*p* < 0.05) was identified between the efficacy of diclofenac (standard) and the tested samples. The anti-inflammatory effects of three ethanol extracts of *A. laxmannii* at different concentrations (25, 50, and 100 mg dw/mL) were evaluated in vivo in a turpentine oil-induced inflammation model in rats by measuring the white blood cell count, differential white blood cell count, total serum nitrite and nitrate levels, total oxidative status, total antioxidant response, and the oxidative stress index. The *A. laxmannii* extracts exhibited anti-inflammatory activity; however, their effects were lower than those of diclofenac for all measured parameters (*p* < 0.001) [19]. Ethanol extracts prepared from the aerial parts of *A. reptans* and *A. genevensis* also exhibited anti-inflammatory effects by reducing oxidative stress, phagocytosis, polymorphonuclear leukocyte (PMN) count, and total leukocyte count in a rat model of acute inflammation induced by a non-antigenic inflammatory stimulus, with diclofenac used as the positive control (*p* < 0.001) [8]. To the best of our knowledge, no published in vitro or in vivo studies have investigated the anti-inflammatory effects of extracts prepared from the aerial parts of *A. chamaepitys*. Therefore, the present study provides the first report of its anti-inflammatory activity.

### 2.4. Tyrosinase Inhibitory Activity

Tyrosinase is the rate-limiting enzyme in melanin synthesis and plays a central role in melanogenesis [39]. In the initial stage of melanin synthesis, tyrosinase catalyzes the hydroxylation of tyrosine to 3,4-dihydroxyphenylalanine (DOPA), followed by the oxidation of DOPA to DOPA quinone. Tyrosinase inhibitors are considered among the most promising agents for inhibiting melanogenesis. Unlike other melanogenesis inhibitors, tyrosinase inhibitors act selectively on melanogenic cells and are therefore associated with fewer adverse effects [39]. The tyrosinase inhibitory activities of the *Ajuga* extracts are presented in Table 2. Overall, the results showed moderate to low tyrosinase inhibitory activity, with IC_50_ values ranging from 4.11 ± 0.24 to 6.88 ± 0.12 mg/mL. Kojic acid, used as the positive control, demonstrated stronger activity than all extracts (IC_50_ = 0.02 mg/mL), with the differences being statistically significant (*p* < 0.05).

Among the tested samples, the strongest tyrosinase inhibitory activity was observed for sample A7 of *A. genevensis*, with an IC_50_ of 4.11 ± 0.24 mg/mL, followed by sample A1 of *A. laxmannii*, with an IC_50_ of 4.59 ± 0.79 mg/mL. In contrast, the lowest inhibitory activity was observed for extract A9 (Table 2). Sample A6 differed significantly (*p* < 0.05) from the other samples in tyrosinase inhibitory activity. Samples A2, A3, and A9 displayed similar inhibitory activity. Additionally, sample A7 differed significantly (*p* < 0.05) from all other samples except A1, A4, and A8. Furthermore, sample A9 showed significantly different tyrosinase inhibitory activity (*p* < 0.05) compared with all other samples except A6. Among the *A. reptans* extracts, sample A6 (Vlasina Plateau) differed significantly (*p* < 0.05) from the extracts collected at the other three localities (Figure 1d). Likewise, all three *A. genevensis* samples collected on the Vlasina Plateau, Suva Planina Mountain, and Vidlič Mountain differed significantly (*p* < 0.05) in their tyrosinase inhibitory activity (Figure 2d).

To evaluate the anti-ageing properties of *A. reptans* root and leaf extracts, tyrosinase inhibition was reported to be 47.52 ± 0.01% and 41.33 ± 0.04%, respectively [40]. However, to the best of our knowledge, there are no published studies on the tyrosinase inhibitory activity of extracts prepared from the aerial parts of these four *Ajuga* species investigated here. Therefore, the present study provides baseline data on their tyrosinase inhibitory activity. Although the constituents of these *Ajuga* species have been recognized as biologically active compounds with potential physiological benefits, their potential applications in cosmetics and functional products remain largely unexplored. Although the precise mechanisms underlying their antioxidant, anti-ageing, and anti-inflammatory effects have not yet been elucidated, these extracts warrant further investigation.

### 2.5. Correlation Analysis Between Phenolic Compound Contents and Examined Activities

**Figure 3:**
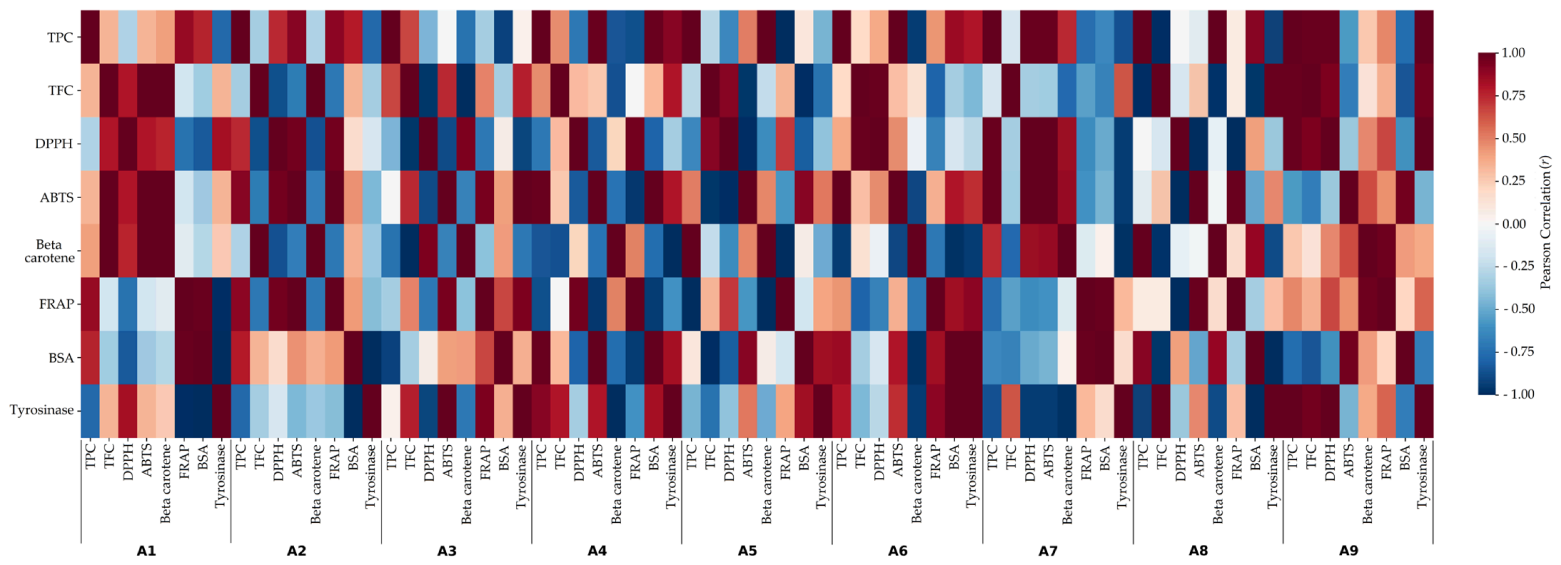
Heatmap of the Pearson correlation matrix showing the relationships among total phenolic content (TPC), total flavonoid content (TFC), antioxidant activity (DPPH and ABTS radical-scavenging, *β*-carotene bleaching, and FRAP assays), anti-inflammatory activity, and tyrosinase inhibitory activity of the *Ajuga* extracts. The blue–white–red color scale ranges from −1 to +1.

Pearson correlation coefficient analysis was conducted to evaluate the relationships among total phenolic content (TPC), total flavonoid content (TFC), and in vitro antioxidant activity (DPPH and ABTS radical-scavenging assays, *β*-carotene bleaching assay, and FRAP assay), as well as anti-inflammatory and tyrosinase inhibitory activities of the *Ajuga* extracts (Figure 3).

In the majority of samples, most prominently highlighted in blocks A1, A3, and A4, a strong positive correlation (dark red) is observed between total phenolic content (TPC) and the FRAP assay. This directly demonstrates that phenolic compounds are the primary contributors to the antioxidant potential through the mechanism of ferric ion reduction. Conversely, these same sample blocks exhibit a pronounced negative correlation (dark blue) between TPC and the DPPH/ABTS radical scavenging assays. Given that a higher antioxidant capacity in DPPH and ABTS protocols is represented by lower numerical outcomes (such as IC_50_ values), this inverse relationship mathematically validates that an increase in phenolic concentration linearly drives a stronger free radical scavenging performance.

Unlike the other groups, samples A7 and A8 display a unique profile where total flavonoid content (TFC) enters into a strong negative correlation with the *β*-carotene bleaching assay. This indicates that within these specific matrix environments, flavonoid structures do not serve as the dominant protectors of lipid systems against peroxidation, pointing instead to the involvement of non-flavonoid constituents or synergistic effects of other secondary metabolites.

The correlation between tyrosinase inhibition and the phytochemical profile varies considerably across the samples. In blocks A2, A5, and A9, a moderate-to-strong positive correlation is visible with both TPC and TFC. This suggests that the phenols and flavonoids present in these specific extracts are directly responsible for suppressing tyrosinase activity, likely acting through copper-chelating mechanisms within the enzyme’s active site or non-competitive pathways.

To evaluate the individual contributions of the 19 identified phenolic compounds to the biological profiles, a Pearson correlation analysis was performed (Table S1). The statistical analysis revealed that specific flavanones (naringin) and phenolic acids (protocatechuic and caffeic acids) predominantly govern the antioxidant capacity of the extracts. Naringin was shown to be the primary driver of antioxidant capacity, showing strong correlations with FRAP (*r* = 0.78), DPPH (*r* = −0.68), and ABTS (*r* = −0.72).

### 2.6. Antimicrobial Activity

#### 2.6.1. Antibacterial Activity

The antibacterial activity of the *Ajuga* extracts is presented in Table 3. The extracts inhibited the growth of the tested Gram-positive and Gram-negative bacterial strains, with minimum inhibitory concentration (MIC) values ranging from 0.12 to 7.5 mg/mL and minimum bactericidal concentration (MBC) values ranging from 0.23 to 15 mg/mL. Among the Gram-positive strains, *Bacillus cereus* was the most susceptible to the *Ajuga* extracts, with MBC values ranging from 0.23 to1.88 mg/mL, whereas among the Gram-negative strains, *Escherichia coli* was the most susceptible, with MBC values ranging from 0.94 to 15 mg/mL. Conversely, the most resistant Gram-positive strains were *Listeria monocytogenes* and *Staphylococcus aureus* (MBCs = 3.15–15 mg/mL), whereas *Enterobacter cloacae* was the most resistant Gram-negative strain (MBCs = 7.5–15 mg/mL). The most active extracts against the tested bacteria were samples A4 and A5 of *A. reptans*, with MBC values ranging from 0.23 to 7.5 mg/mL for A4 and from 0.23 to 15 mg/mL for A5. In contrast, samples A1 and A9 were the least effective against six of the bacterial strains tested. For sample A1, MBC values corresponding to the highest tested concentration were observed against *B. cereus* and *Salmonella typhimurium* (Table 3).

The antimicrobial activity observed in the present study generally agrees with previous reports demonstrating that *Ajuga* species exhibit greater activity against Gram-positive than Gram-negative bacteria. Among the investigated extracts, *A. reptans* exhibited the strongest antibacterial activity, whereas *A. laxmannii* showed the highest antifungal activity. Similar trends have been reported by Toiu et al. [8,22], who demonstrated that ethanol extracts of *A. genevensis*, *A. reptans*, and *A. laxmannii* were particularly effective against *Staphylococcus aureus*. However, the MIC and MBC values obtained in the present study differed from those reported previously, possibly due to differences in extraction procedures, plant origin, environmental conditions, and the phytochemical composition of the extracts.

Our findings are also partially consistent with those of Göger et al. [28], who reported antimicrobial activity of methanolic extracts prepared from the aerial parts of *A. reptans* and *A. genevensis* against *Escherichia coli*, *Staphylococcus aureus*, *Salmonella typhimurium*, and *Bacillus cereus*. Although the absolute MIC values differed from those obtained in the present study, both studies confirmed the antimicrobial potential of these species. Likewise, *S. typhimurium* and *Listeria monocytogenes* were among the least susceptible bacterial strains, consistent with the findings of Toiu et al. [8,22].

Previous studies using the disk diffusion method also demonstrated the antibacterial activity of *A. reptans* extracts against several bacterial species, including *Bacillus subtilis*, *Serratia marcescens*, *Pseudomonas aeruginosa*, *Proteus vulgaris*, *Enterobacter cloacae*, and *Staphylococcus epidermidis* [41,42,43]. Although direct comparison between disk diffusion and broth microdilution assays is limited because the two methods evaluate antimicrobial activity using different principles, both consistently confirm the antibacterial properties of *Ajuga* extracts.

The stronger activity against Gram-positive bacteria observed in the present study is further supported by previous investigations of *A. chamaepitys* subsp. *chamaepitys*. Acetone, methanol, and ethyl acetate extracts of this subspecies exhibited pronounced antibacterial activity against *Bacillus cereus*, *Bacillus subtilis*, *Bacillus pumilus*, and *Staphylococcus aureus*, whereas their activity against Gram-negative bacteria was considerably weaker [39]. These findings are consistent with our results and suggest that Gram-positive bacteria are generally more susceptible to *Ajuga* extracts, likely due to structural differences in the bacterial cell envelope that facilitate the penetration of phenolic compounds.

Overall, despite quantitative differences in antimicrobial activity, the present findings are consistent with the published literature in indicating that *Ajuga* species represent promising natural sources of antimicrobial compounds, particularly against Gram-positive bacteria.

Based on the Pearson correlation (Table S1), it was shown that the antimicrobial activity (MIC) was strongly driven by p-hydroxybenzoic acid and caffeic acid. Specifically, p-hydroxybenzoic acid correlated strongly with the inhibition of *M. flavus* (*r* = −0.73) and *E. coli* (*r* = −0.68), while caffeic acid was highly effective against *M. flavus* (*r* = −0.71) and *L. monocytogenes* (*r* = −0.72). Rosmarinic acid also actively contributed to anti-*S. aureus* efficiency (*r* = −0.66).

#### 2.6.2. Antifungal Activity

The antifungal activity of the *Ajuga* extracts tested is shown in Table 4.

The minimum inhibitory concentration (MIC) values ranged from 0.06 to 22.50 mg/mL, while the minimum fungicidal concentration (MFC) values ranged from 0.23 to >15 mg/mL. Overall, the extracts exhibited lower inhibitory activity against the tested fungi than against the bacterial strains. The most promising antifungal extract was *A. laxmannii* (A1), with MFC values ranging from 0.23 to >15 mg/mL, although it was the least active against bacteria. Sample A8 inhibited fungal growth only at the highest concentrations tested (MFC > 15 mg/mL for all fungal strains), followed by sample A7, which showed similar activity, with MFC values ranging from 7.50 to >15 mg/mL (Table 4). *Penicillium funiculosum* was the most susceptible fungal species, with MFC values ranging from 0.23 to 7.50 mg/mL, except for sample A8 (MFC > 15 mg/mL), whereas *Aspergillus niger* and *A. ochraceus* were the most resistant species (MFC > 15 mg/mL for both; Table 4). The antifungal activity observed in the present study is generally consistent with previous reports demonstrating that *Ajuga* species exhibit pronounced activity against several pathogenic fungi. Among the investigated species, *A. laxmannii* exhibited the strongest antifungal activity, whereas *A. reptans* and *A. genevensis* showed moderate activity depending on the fungal species tested.

Our findings are in partial agreement with those of Toiu et al. [22], who reported that ethanol extracts of *A. laxmannii* exhibited remarkable antifungal activity, particularly against *Candida parapsilosis* (MIC = 0.012 mg/mL; MFC = 0.025 mg/mL). The same authors also demonstrated strong fungicidal activity of chloroform extracts of *A. laxmannii* against *Candida albicans* and *Penicillium funiculosum*. Although the absolute MIC and MFC values obtained in the present study differed from those previously reported, both studies confirm the high antifungal potential of *A. laxmannii* extracts.

Similarly, Toiu et al. [8] showed that petroleum ether, chloroform, and ethanol extracts of *A. reptans* exhibited strong activity against *Candida albicans*, whereas *P. funiculosum* and *Aspergillus niger* were among the least susceptible fungal species to *A. genevensis* extracts. In contrast, *P. funiculosum* was one of the most susceptible fungal species in the present study. These differences are likely attributable to variations in the extraction solvent, geographical origin of the plant material, environmental conditions, and the resulting phytochemical composition of the extracts.

To the best of our knowledge, this is the first study to evaluate the antimicrobial and antifungal activities of *A. chamaepitys*. Therefore, direct comparison with previous reports is not possible. Nevertheless, the present results indicate that this species possesses measurable antimicrobial potential and provide baseline data for future phytochemical and pharmacological investigations.

Regarding the antifungal activity, the Pearson correlation analysis revealed highly compound-specific screening profiles (Table S1). A remarkably strong negative correlation was observed between vitexin content and the minimum inhibitory concentrations (MIC) against *Penicillium funiculosum* (*r* = −0.76). This robust mathematical correlation explains why the *A. laxmannii* extract (sample A1), which possessed by far the highest quantified concentration of vitexin (1.51 mg/g), demonstrated the most potent suppressive activity against this fungal strain (MIC = 0.06 mg/mL). Furthermore, naringin content was found to heavily drive the efficacy against *Trichoderma viride*, showing a significant negative correlation coefficient (*r* = −0.74). This finding is strongly supported by the experimental data, where sample A3 (*A. reptans* from Vidlič Mtn.), characterized by the highest naringin concentration (2.54 mg/g), exerted the lowest MIC values against *T. viride*. Additionally, rosmarinic acid demonstrated notable selective antifungal properties, correlating significantly with the inhibition of *Penicillium verrucosum* (*r* = −0.63) and *P. ochrochloron* (*r* = −0.61).

### 2.7. Inhibition of Pyocyanin Production in Pseudomonas aeruginosa PAO1

Among all investigated biological activities, the inhibition of pyocyanin production was one of the most notable findings.

Pyocyanin production by *P. aeruginosa* was evaluated in the presence of *Ajuga* extracts at one-half of their respective MIC values (Table 3). The synthesis of this toxin varied according to the inhibitory potential of the tested samples (A1–A9), with the results presented in Table 5.

Pyocyanin synthesis was most effectively inhibited by extracts A9 and A7, allowing only 60.2% and 68.34% of pyocyanin production, respectively. The remaining extracts inhibited pyocyanin production in the following decreasing order: A1 > A2 > A8. In contrast, all *A. reptans* extracts A3, A4, A5, and A6 did not exhibit detectable inhibitory activity in this assay. These extracts, as well as ampicillin, had enhancing effects on pyocyanin production. The opposite effects of *A. reptans* and *A. genevensis* (A7, A8 and A9) extracts on pyocyanin production could be explained by differences in their phytochemical composition. *A. genevensis* extracts suppressed pyocyanin production, which is consistent with their relatively higher content of phenolic compounds: *p*-coumaric acid (0.41–0.71 mg/kg), ferulic acid (1.25–2.14 mg/kg), and flavones: luteolin (0.55–0.73 mg/kg), and apigenin (0.72–0.94 mg/kg), which have been reported to inhibit quorum-sensing (QS)-regulated virulence in *P. aeruginosa* [44,45]. In addition, synergistic interactions between metabolites play an important role in mediating the pyocyanin-suppressive effect.

The stimulatory effect of all *A. reptans* extracts could be attributed to the significantly lower concentrations of these compounds (*p*-coumaric acid, 0–0.33 mg/kg; ferulic acid, 0.61–1.20 mg/kg; luteolin, 0.20–0.25 mg/kg; apigenin, 0.14–0.18 mg/kg). It is also possible that extracts A3–A6 increase the expression of virulence factors when used at sub-inhibitory concentrations, eliciting a bacterial adaptive response. Therefore, variations in the qualitative and quantitative chemical profiles of extracts from *A. repans* and *A. genevensis* may have differing effects on the bacterial signaling pathways involved in the manufacture of pyocyanins.

**Figure 4:**
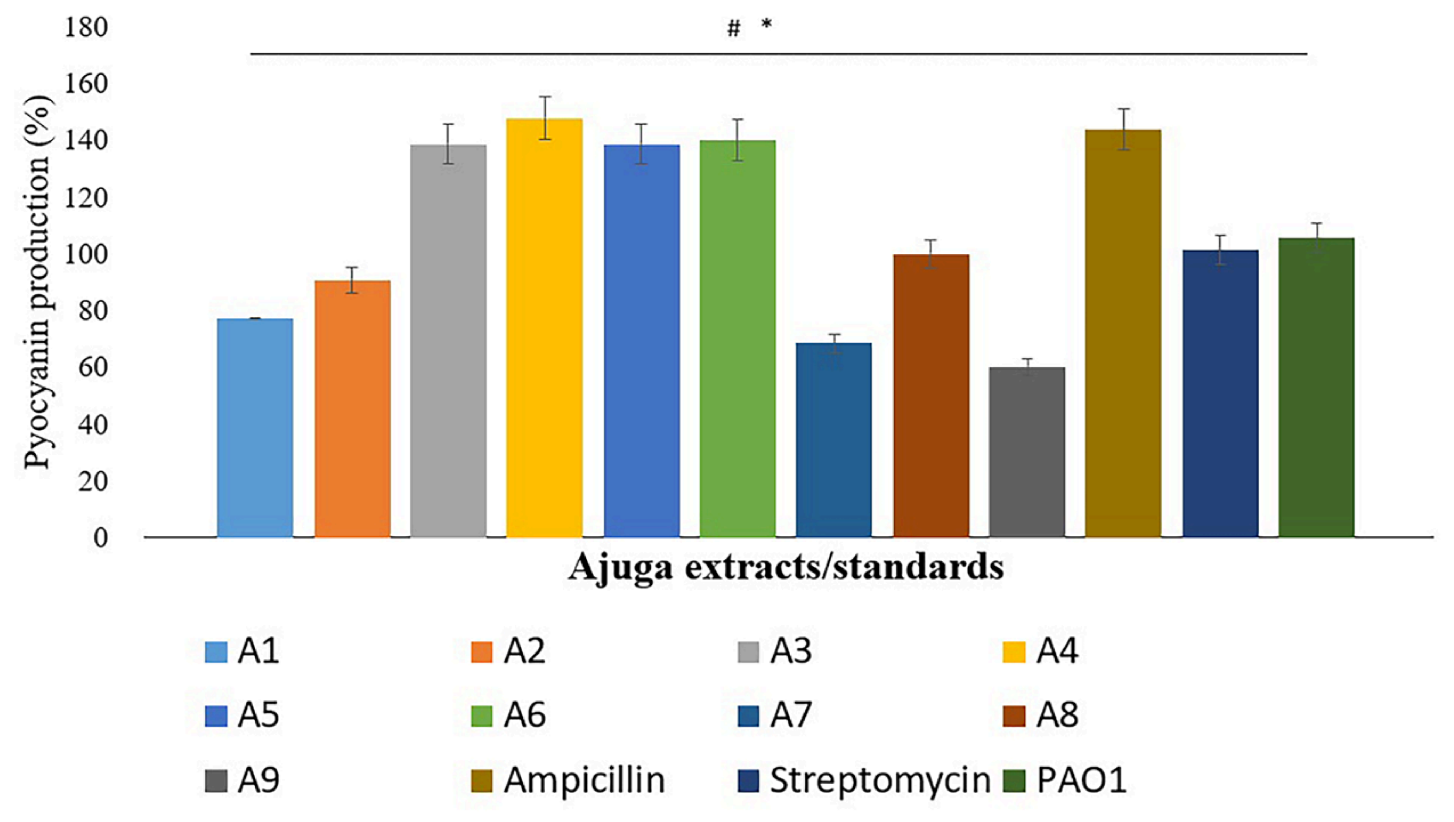
Effect of *Ajuga* extracts from different collection localities on pyocyanin production by *Pseudomonas aeruginosa* PAO1. *Ajuga laxmannii*: A1—Vidlič Mountain; *Ajuga chamaepitys:* A2—Vidlič Mountain; *Ajuga reptans*: A3—Vidlič Mountain, A4—Suva Planina Mountain, A5—Rtanj Mountain, A6—Vlasina Plateau; *Ajuga genevensis*: A7—Vlasina Plateau, A8—Suva Planina Mountain, A9—Vidlič Mountain. Positive controls: ampicillin and streptomycin; negative control: PAO1. Data are presented as the mean ± SD and were analyzed using one-way ANOVA followed by Tukey’s post hoc test. # *p* < 0.05 (# indicates significant differences among samples); * *p* < 0.05 (* indicates significant differences between the samples and the corresponding standards).

Compared with the antibiotics ampicillin and streptomycin, five of the nine tested extracts were more effective at inhibiting pyocyanin production (Table 5). Furthermore, statistically significant differences (*p* < 0.05) were observed between the activities of the reference antibiotics and those of the tested samples (Figure 4). To the best of our knowledge, no previous studies have evaluated the inhibition of pyocyanin production by *A. chamaepitys*, *A. genevensis*, *A. laxmannii*, or *A. reptans*.

Further studies are therefore necessary to elucidate in detail the activity of the tested extracts, including comprehensive metabolomics profiling and analysis of QS-regulated gene expression, in order to identify the metabolites responsible for pyocyanin production, establish concentration ranges that avoid undesirable stimulation of bacterial virulence, and determine their safety as potential antimicrobial agents.

Although the antioxidant and antimicrobial activities of *Ajuga* species have been extensively reported, their influence on pyocyanin production has received very limited attention [40]. In the present study, *A. genevensis* extracts inhibited pyocyanin production approximately twice as effectively as ampicillin, representing one of the most notable biological activities observed among all the tested extracts.

This finding substantially expands the known biological profile of *Ajuga* species and suggests that Serbian populations of *A. genevensis* may constitute a valuable natural source of antivirulence compounds [46]. Pyocyanin biosynthesis is primarily regulated by the quorum-sensing network of *P*. *aeruginosa* [47]. Although the molecular mechanism was not investigated in the present study, the observed inhibition of pyocyanin production at subinhibitory concentrations without evidence of complete bacterial growth inhibition suggests that the active constituents may interfere with quorum-sensing signaling pathways or other regulatory mechanisms controlling virulence factor production rather than exerting a direct bactericidal effect [48,49]. This hypothesis, however, requires experimental confirmation. While the responsible metabolites remain to be identified, the observed activity suggests that inhibition of bacterial virulence, rather than bacterial growth, may represent an additional pharmacological mechanism of *Ajuga* extracts.

The present study has several limitations that should be considered when interpreting the results. All biological activities were evaluated exclusively under in vitro conditions; therefore, the observed effects cannot be directly extrapolated to in vivo systems. In addition, although the phytochemical profiles of the extracts were comprehensively characterized, no bioactivity-guided fractionation or mechanistic studies were performed to identify the compounds responsible for the observed biological activities, particularly the pronounced anti-pyocyanin effect.

Future studies should combine bioactivity-guided fractionation with transcriptomic and molecular approaches to identify the compounds responsible for the observed anti-pyocyanin activity and determine whether they interfere with quorum-sensing pathways or other virulence-regulating mechanisms. Furthermore, in vivo studies and toxicity assessments will be essential to confirm the biological relevance, efficacy, and safety of these extracts before considering their pharmaceutical or food-related applications.

Overall, the results obtained demonstrate considerable interspecific variability among the investigated *Ajuga* species. The differences observed in phytochemical profiles and biological activities are likely attributable to both species-specific characteristics and environmental conditions of the collection localities. These findings further emphasize the importance of geographical origin when evaluating medicinal plant resources.

## 3. Materials and Methods

### 3.1. Plant Material and Extract Preparation

**Figure 5:**
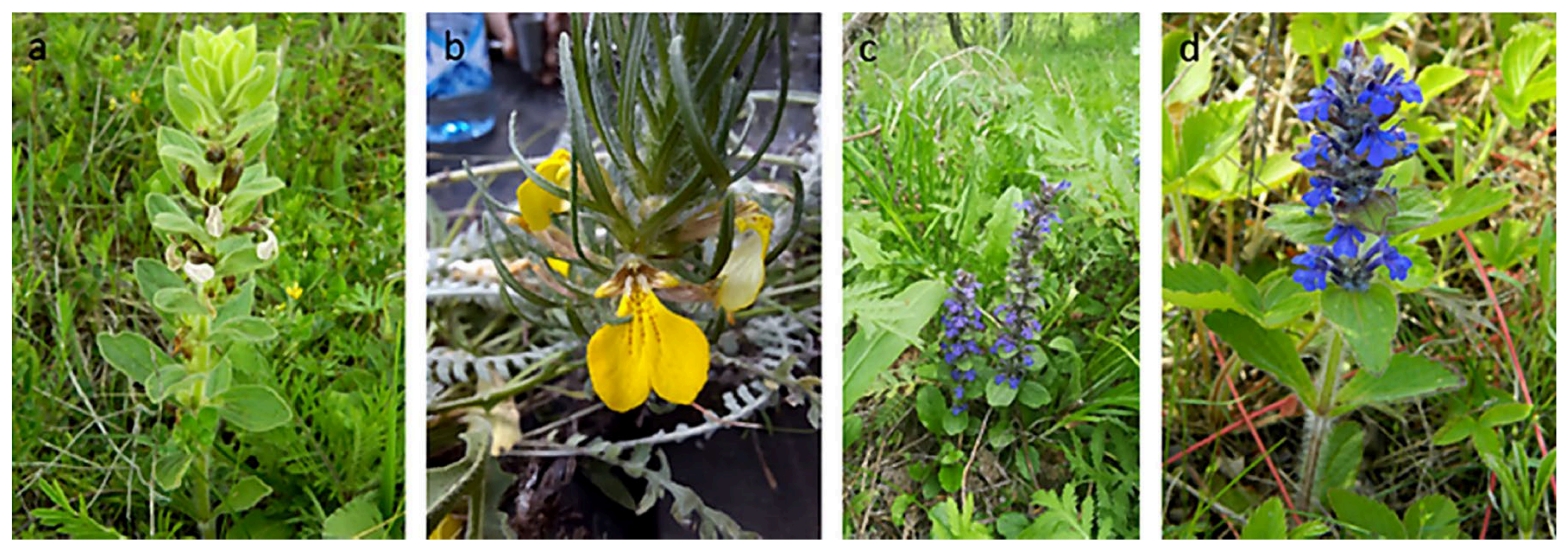
Natural habitat and aerial parts of *Ajuga laxmannii* (**a**), *A. chamaepitys* (**b**), *A. reptans* (**c**) and *A. genevensis* (**d**) from Serbia.

The aerial parts of wild-growing plants were collected throughout Serbia in July 2018 (Figure 5).

The plants were identified by Marija Marković, and voucher specimens were deposited in the Herbarium of the Faculty of Sciences and Mathematics, University of Niš, Serbia. The collected specimens are listed in Table 6. Plant material (10 g) was homogenized and extracted with 70% (*v*/*v*) ethanol. Organic solvents (p.a.) were purchased from Zorka Pharma Šabac, Serbia. Extraction was carried out under reduced-light conditions for 24 h, with the samples exposed to an ultrasonic bath (Sonic 8GT, Serbia) during the first and last hours. The extracts were filtered through Whatman No. 1 filter paper and concentrated under reduced temperature and pressure (Büchi R-114 Rotary evaporator with Büchi B-480 Water Bath, Switzerland). Extraction yields were expressed as mg of dry extract per gram of dry plant material (mg/g dw) (Table 6) [50]. 

### 3.2. Phytochemical Composition

The measurements were performed using a Jenway 6305 UV-Vis spectrophotometer (Bibby Scientific Ltd., Stone, Staffordshire, UK) against an appropriate blank. The total phenolic content (TPC) and total flavonoid content (TFC) of the extract solutions (1 mg/mL) were expressed as gallic acid equivalents (GAE) and quercetin hydrate equivalents (QuE) per gram of dry extract, respectively. Folin-Ciocalteu phenol reagent (FC), formic acid (FA), 2,2-diphenyl-1-picrylhydrazyl (DPPH), bovine serum albumin (BSA), 2(3)-tbutyl-4-hydroxyanisole (BHA), gallic acid and vitamin C (ascorbic acid) were obtained from Sigma Aldrich Co. (St. Louis, MO, USA). 2,2′-azino-bis(3-ethylbenzothiazoline)-6-sulfonic acid (ABTS) was purchased from Merck (Darmstadt, Germany). Potassium acetate, potassium peroxydisulfate, and anhydrous sodium carbonate were purchased from AnalaR Normapur, VWR, Geldenaaksebaan, Leuven Belgium, while aluminum nitrate nonahydrate was obtained from Fluka Chemie AG, Buchs, Switzerland. Quercetin hydrate was obtained from TCI Europe N.V. (Zwijndrecht, Belgium).

#### 3.2.1. Ultra-High-Performance Liquid Chromatography-Mass Spectrometry Analysis of Phenolic Compounds (UHPLC-MS/MS)

The chemical characterisation of the phenolic compounds in the *Ajuga* extracts was preceded by dissolving the dry extracts in MeOH (20 mg/mL) and filtering through a 0.45 μm syringe filter. Methanolic stock solutions of the commercial standards were prepared at final concentrations of 0.025–1.000 μg/mL. Analysis of the target compounds involved separation, identification, and quantification of phenols using a Dionex Ultimate 3000 ultra-high-performance liquid chromatography (UHPLC, Thermo Fisher Scientific, Bremen, Germany) system with a triple quadrupole (QqQ) mass spectrometer (TSQ Quantum Access Max, Thermo Fisher Scientific, Basel, Switzerland). The analysis was performed according to previously described experimental chromatographic conditions and mass spectrometry parameters, with some modification [51].

Elution was performed at 40 °C on a Hypersil gold C18-column (50  ×  2.1 mm) with 1.9 µm particle size (ThermoFisher Scientific, Fair Lawn, NJ, USA). The mobile phase consisted of (A) water with 0.1% formic acid and (B) acetonitrile (MS grade, Fisher Scientific, Loughborough, UK) with 0.1% formic acid, which were applied in the following gradient elution: 5% B in the first min; 5–95% B from 1 to 10 min; 95% B from 10 to 12 min; 5% B until 15 min. The flow rate was set to 0.3 mL/min, and the injection volume was 5 μL.

A TSQ Quantum Access Max QQQ mass spectrometer equipped with a HESI source was used with vapouriser temperature kept at 450 °C and ion source settings as follows: spray voltage 4000 V, sheath gas (N_2_) pressure 50 AU, ion sweep gas pressure 0 AU and auxiliary gas (N_2_) pressure 20 AU, capillary temperature at 320 °C, skimmer offset 0 V. The MS data were acquired in negative mode, in the *m/z* range from 100 to 1000. A selected reaction monitoring (SRM) experiment for quantitative analysis was performed using two MS^2^ fragments for each compound, which were previously defined as dominant in product ion scan experiments.

Data acquisition and analysis were performed using Xcalibur software (2.1). Phenolic compounds were identified and quantified by comparison with commercial standards, and the results were expressed as mg/kg dry extract [52].

#### 3.2.2. Total Phenolic Content (TPC)

The TPC was determined using a modified method described by Singleton et al. (1999) [53]. Quantitative measurements were performed using 10% (*v*/*v*) Folin–Ciocalteu reagent. The reaction mixture, consisting of 0.1 mL extract solution and 1 mL of the Folin–Ciocalteu reagent, was supplemented after 6 min of incubation with 0.8 mL of 7.5% (*w*/*v*) sodium carbonate. The samples were incubated for 2 h, and the absorbance was measured at 740 nm.

#### 3.2.3. Total Flavonoid Content (TFC)

The TFC of the extracts was determined by the method of Mileski et al. (2017) [54] with slight modifications. Flavonoid content was quantified using a reagent mixture of 80% (*v*/*v*) ethanol, 10% (*w*/*v*) aluminum nitrate, and 1 M (*w*/*v*) potassium acetate. A 0.6 mL aliquot of the extract solution was mixed with 0.58 mL of reagent mixture, and after 40 min of incubation, absorbance was measured at 415 nm.

### 3.3. Antioxidant Activity

Spectrophotometric measurements were performed using a Jenway 6305 UV-Vis spectrophotometer against an appropriate blank. For the DPPH, ABTS, and *β*-carotene bleaching assays, the results were expressed as IC_50_ values (mg/mL), defined as the extract concentration required to achieve 50% antioxidant activity. For the FRAP assay, antioxidant activity was expressed as μmol Fe^2+^ equivalents per mg of dry extract, using a calibration curve constructed with ferrous sulfate heptahydrate. BHA and ascorbic acid were used as reference standards.

#### 3.3.1. DPPH Assay

The free radical scavenging activity was evaluated using the 2,2-diphenyl-1-picrylhydrazyl (DPPH) assay [55]. Briefly, 0.1 mL of the methanolic extract solution was added to 0.9 mL of a methanolic DPPH solution (0.04 mg/mL). The reaction mixture was incubated in the dark at room temperature for 30 min, and the absorbance was measured at 517 nm. The DPPH radical scavenging activity was calculated using the following equation (Equation (1)):

I (%) = (A_c_ − A_s_) × 100/A_c_ (1)

where A_c_ is the absorbance of the control, and A_s_ is the absorbance of the sample.

#### 3.3.2. ABTS Assay

The 2,2′-azino-bis (3-ethylbenzothiazoline-6-sulfonic acid) (ABTS) radical-scavenging assay was performed according to the method of Miller and Rice-Evans (1997) [56], with slight modifications. The ABTS•+ stock solution was prepared by mixing 19.2 mg of ABTS with 5 mL of 2.46 mM potassium persulfate. After 12–16 h, 1 mL of the ABTS•+ solution was diluted with 100 mL of deionized water (dH_2_O) to obtain an absorbance of 0.7 ± 0.02 at 734 nm. Briefly, 25 μL of the extract solution was mixed with 1 mL of the diluted ABTS•+ solution and incubated at 30 °C for 30 min. The absorbance was measured at 734 nm. The ABTS•+ radical-scavenging activity was calculated using the following equation (Equation (2)):

I (%) = (A_c_ − A_s_) × 100/A_c_ (2)

where A_c_ is the absorbance of the control, and A_s_ is the absorbance of the sample.

#### 3.3.3. β-Carotene (βC) Bleaching Assay

The antioxidant activity was measured using the *β*-carotene bleaching assay according to the method described by [57], with slight modifications. The *β*-carotene-linoleic acid emulsion was prepared from a *β*-carotene solution in chloroform (5 mg/mL), linoleic acid (20 mg), and Tween-40 (200 mg). After evaporation of the chloroform, the mixture was emulsified with 50 mL of oxygenated water. Aliquots (0.1 mL) of the extract solutions were mixed with 2.4 mL of the emulsion and incubated at 50 °C for 2 h. The absorbance of the samples and control was measured at 470 nm at the beginning (A_s0_, A_c0_) and after incubation (A_s120_, A_c120_). The inhibition of *β*-carotene bleaching was calculated using the following equation (Equation (3)):

I (%) = [((Ac_0_ – Ac_120_) – (As_0_ – As_120_))/(Ac_0_ – Ac_120_)] × 100 (3)

#### 3.3.4. FRAP Assay

Ferric reducing antioxidant power was measured using the FRAP assay as described by [58], with slight modifications. The FRAP working reagent was prepared by mixing 300 mM acetate buffer (pH 3.6), 10 mM TPTZ (2,4,6-tripyridyl-s-triazine) in 40 mM HCl, and 20 mM FeCl_3_ in a 10:1:1 ratio. The plant extract (0.15 mL) was mixed with the FRAP reagent (2.85 mL) and incubated at room temperature in the dark for 30 min. Absorbance was measured spectrophotometrically at λmax = 593 nm.

### 3.4. Protein Denaturation

The in vitro anti-inflammatory activity was evaluated using the bovine serum albumin (BSA) denaturation assay. The inhibitory effect of *Ajuga* extracts on BSA denaturation was evaluated using diclofenac sodium (0.1 mg/mL) as the reference standard, according to the method of Rahman et al. (2015) [59]. Aliquots (0.05 mL) of the extract solutions (0.1 mg/mL) were mixed with 0.45 mL of BSA (50 mg/mL) and incubated at 37 °C for 20 min. The reaction mixtures were then heated to 57 °C for 3 min, followed by the addition of 2.5 mL of 0.5 M phosphate buffer (pH 6.3). The absorbance was measured at 255 nm, and the results were expressed as the percentage inhibition of BSA denaturation, calculated using the following equation (Equation (4)):

Protein denaturation (%) = 100 − ((optical density of test solution − optical density of sample blank)/optical density of test control) × 100)(4)

### 3.5. Tyrosinase Inhibitory Activity

Tyrosinase inhibitory activity was evaluated using the modified dopachrome method with L-DOPA (L-3,4-dihydroxyphenylalanine) as the substrate [60]. Briefly, 0.025 mL of the sample solution in phosphate-buffered saline (PBS) was mixed with 0.1 mL of 25 mM phosphate buffer (pH 6.8) and 0.04 mL of tyrosinase solution (200 U/mL) in a 96-well microplate and incubated at 25 °C for 15 min. The reaction was initiated by adding 0.04 mL of 2.5 mM L-DOPA solution prepared in phosphate buffer, followed by incubation at 25 °C for 10 min. The absorbance was measured at 492 nm using a Thermo Fisher Scientific Multiskan Sky microplate reader. Tyrosinase inhibitory activity was calculated using Equation (1). The results were expressed as IC_50_ values (mg/mL), defined as the extract concentrations required to achieve 50% inhibition of tyrosinase activity, and were compared with kojic acid as the reference standard.

### 3.6. Antimicrobial Activity

#### 3.6.1. Antibacterial Activity

For the determination of antibacterial activity, the following Gram-positive and Gram-negative bacterial strains were used: *Staphylococcus aureus* (ATCC 11632), *Micrococcus flavus* (ATCC 10240), *Bacillus cereus* (ATCC 10876), *Listeria monocytogenes* (NCTC 7973), *Salmonella typhimurium* (ATCC 13311), *Escherichia coli* (ATCC 25922), *Enterobacter cloacae* (ATCC 35030), and *Pseudomonas aeruginosa* PAO1.

MIC and MBC were determined using the 96-well microdilution method as previously described [61,62]. Extracts dissolved in 30% (*v*/*v*) ethanol (negative control) at a concentration of 30 mg/mL were added to tryptic soy broth (TSB) and inoculated with bacteria to a final concentration of 1 × 10^6^ colony-forming units (CFU)/well. The MIC was defined as the lowest extract concentration (mg/mL) that inhibited at least 50% of bacterial growth compared with the positive control. The MBC was defined as the lowest extract concentration (mg/mL) that completely inhibited visible bacterial growth after subculturing the treated bacteria. Ampicillin and streptomycin (1 mg/mL, Sigma-Aldrich, St. Louis, MO, USA) were used as positive controls.

#### 3.6.2. Antifungal Activity

The antifungal activity was evaluated against the following fungal strains, obtained from the Mycological Laboratory, Department of Plant Physiology, Institute for Biological Research “Siniša Stanković” National Institute of Republic of Serbia, Belgrade, as follows: *Aspergillus fumigatus* (ATCC 204305), *Aspergillus versicolor* (ATCC 11730), *Aspergillus ochraceus* (ATCC 12066), *Aspergillus niger* (ATCC 6275), *Trichoderma viride* (IAM 5061), *Penicillium funiculosum* (ATCC 36839), *P. ochrochloron* (ATCC 9112) and *P*. *verrucosum* var. *cyclopium* (Pvc-DS-11). A modified microdilution method was used to determine antifungal activity [63]. Fungal spores were harvested from the surface of agar plates with sterile 0.85% (*w*/*v*) saline containing 0.1% (*v*/*v*) Tween 80. The spore suspension was then adjusted with sterile saline to a concentration of approximately 1.0 × 10^5^ spores/100 μL per well. Dilutions of the inoculum were cultured on malt extract agar to verify the absence of contamination and confirm the validity of the inoculum. Tested *Ajuga* extracts were dissolved in 30% (*v*/*v*) ethanol to a final concentration of 30 mg/mL and added to malt broth containing the final inoculum. MICs were determined using the serial microdilution method in 96-well microtiter plates, and the lowest extract concentrations showing no visible fungal growth under a binocular microscope were recorded as the MICs. MFCs were determined by subculturing 2 μL from each well showing no visible growth onto fresh microtiter plates containing 100 μL of malt broth per well, followed by incubation for 72 h at 28 °C. The lowest extract concentrations showing no visible fungal growth after subculturing were recorded as the MFCs, corresponding to 99.5% killing of the original inoculum. Thirty percent (30%, *v*/*v*) ethanol was used as the negative control, whereas ketoconazole and nystatin (both 1 mg/mL, Sigma-Aldrich, St. Louis, MO, USA) were used as positive controls.

### 3.7. Anti-Pyocyanin Test

All nine *Ajuga* extracts were evaluated for their ability to inhibit pyocyanin production by *Pseudomonas aeruginosa* PAO1, obtained from the collection of the Institute for Biological Research “Siniša Stanković”, National Institute of the Republic of Serbia, Belgrade, Serbia. This strain was cultured in Luria–Bertani (LB) broth (1% *w/v* NaCl, 1% *w/v* tryptone, 0.5% *w/v* yeast extract) at 37 °C with shaking at 220 rpm [64]. Pyocyanin production was assessed using the flask assay after treatment with the extracts at 0.5 × MIC. The extracts were dissolved in 30% (*v*/*v*) ethanol at an initial concentration of 30 mg/mL. Briefly, 5 mL of overnight PAO1 culture was adjusted to an OD_600_ nm of 0.2 and mixed with extract solutions. After incubation at 37 °C for 24 h, the culture was extracted with 3 mL of chloroform, and the chloroform phase was subsequently re-extracted with 1 mL of 0.2 M HCl. The absorbance of the aqueous (upper) phase was measured at 520 nm using a Shimadzu UV1601 spectrophotometer (Shimadzu Corporation, Kyoto, Japan). Pyocyanin production (%) was calculated using the following equation:

(OD_600_ − OD_520_)/OD_600_) × 100.

### 3.8. Statistical Analysis

All experiments were performed in triplicate and repeated three independent times. The results obtained are presented as mean ± standard deviation (SD). Statistical analysis was performed using one-way ANOVA, followed by Tukey’s post hoc test in SPSS v. 20.0. Differences were considered statistically significant at *p* < 0.05. Pearson’s linear correlation coefficient was used for the correlation analysis. The heatmaps for the antioxidant activity test were generated using OriginPro 2021 (OriginLab Corporation, Northampton, MA, USA) graphing and data analysis software.

Multivariate data analysis and visualization were performed using Python programming language (version 3.10). Prior to the analyses, raw numerical data were preprocessed by extracting mean values and handling missing values where appropriate. Principal Component Analysis (PCA) was executed on the quantified phenolic compounds data matrix using the scikit-learn package. Data standardization (autoscaling to unit variance) was applied before PCA decomposition to prevent variables with larger absolute scales from dominating the analysis.

Hierarchical cluster analysis (HCA) and heatmaps were constructed using the seaborn and matplotlib libraries. For the clustered heatmap, Z-score normalization was applied to the biological activity variables across the samples to ensure comparability of different analytical scales, and clustering was performed using the Euclidean distance metric and the complete linkage method. Non-linear regression analysis for the dose–response kinetics was modeled via a four-parameter logistic (Hill) equation using numpy and plotted with matplotlib to determine and compare the mathematical fit of the top-performing extracts.

## 4. Conclusions

In conclusion, the ethanol extracts of selected *Ajuga* species collected from different localities in Serbia exhibited distinct phytochemical profiles and diverse biological activities, demonstrating that both species identity and geographical origin markedly influence their biological potential. UHPLC-MS/MS analysis identified 19 phenolic compounds, with *p*-hydroxybenzoic acid and ferulic acid being the predominant constituents in most extracts. *A. reptans* (A3, Vidlič Mountain) contained the highest total phenolic content (120.52 mg GAE/g dw) and exhibited the strongest antioxidant activity in the DPPH, ABTS, β-carotene bleaching, and FRAP assays. *A. laxmannii* (A1) contained the highest flavonoid content (48.95 mg QuE/g dw) and exhibited the strongest antifungal activity. *Penicillium funiculosum* was the most susceptible fungal species, whereas *Aspergillus niger* and *A. ochraceus* were the most resistant. *A. reptans* extracts from Rtanj Mountain (A5), Suva Planina Mountain (A4), and Vidlič Mountain (A3) exhibited the strongest anti-inflammatory activity, whereas *A. genevensis* (A7) showed the highest tyrosinase inhibitory activity. Among the tested bacteria, the strongest antibacterial activity was exhibited by *A. reptans* extracts (A4 and A5), whereas the most notable finding of this study was the pronounced anti-pyocyanin activity of *A. genevensis* extracts (A7 and A9), which inhibited pyocyanin production approximately twice as effectively as ampicillin. This finding expands the currently known biological profile of *Ajuga* species by demonstrating their promising antivirulence potential against *Pseudomonas aeruginosa* and represents the principal novel contribution of the present study.

The obtained results provide a foundation for future bioactivity-guided isolation and structural characterization of the compounds responsible for anti-pyocyanin activity, followed by mechanistic studies of quorum-sensing inhibition and in vivo evaluation of efficacy and safety. In addition, the demonstrated antioxidant, antimicrobial, antifungal, anti-inflammatory, and antivirulence properties support further investigation of Serbian *Ajuga* species as potential sources of phytopharmaceuticals, functional food ingredients, and natural preservatives.

## Figures and Tables

**Table 1:** The phytochemical constituents of the *Ajuga* extracts (mg/kg). Sample codes: A1—*A. laxmannii* (Vidlič Mountain); A2—*A. chamaepitys* (Vidlič Mountain); A3—*A. reptans* (Vidlič Mountain); A4—*A. reptans* (Suva Planina Mountain); A5—*A. reptans* (Rtanj Mountain); A6—*A. reptans* (Vlasina Plateau); A7—*A. genevensis* (Vlasina Plateau); A8—*A. genevensis* (Suva Planina Mountain); A9—*A. genevensis* (Vidlič Mountain). NF—not found.

Class of Compounds	Compound	A1	A2	A3	A4	A5	A6	A7	A8	A9
Hydroxybenzoic acids	Protocatechuic_acid	0.30	0.48	0.29	0.27	0.15	0.35	0.29	0.23	0.20
	*p*-Hydroxybenzoic acid	0.72	1.78	NF	1.42	1.80	1.76	1.30	1.31	1.24
	Gentisic acid	NF	0.27	0.21	0.21	0.25	NF	NF	0.17	0.38
Hydroxycinnamic acids	Caffeic acid	0.25	0.87	0.86	1.41	1.08	0.87	0.96	0.91	0.88
	*p*-Coumaric acid	0.34	0.27	NF	0.33	NF	0.28	0.71	0.41	0.71
	5-*O*-Caffeoylquinic acid	0.47	NF	1.02	NF	2.48	1.22	0.57	NF	NF
	Ferulic acid	1.16	5.81	0.61	1.04	0.95	1.20	2.00	1.25	2.14
	Rosmarinic acid	0.38	0.20	0.11	0.08	0.08	0.10	0.05	0.29	0.03
Flavones	Luteolin	0.08	1.55	0.22	0.25	0.20	0.20	0.55	0.73	0.56
	Apigenin	0.04	0.34	0.14	0.14	0.15	0.18	0.80	0.72	0.94
	Hispidulin	0.03	0.08	0.02	0.02	0.03	0.04	0.11	0.08	0.14
	Cirsimaritin	0.07	0.02	NF	NF	NF	0.02	0.04	0.02	NF
Flavone glycoside	Vitexin	1.51	0.31	0.02	0.02	0.02	0.03	0.04	0.07	0.04
Flavanones	Eriodictyol	0.03	0.03	0.02	0.02	0.02	0.03	0.03	0.03	0.03
	Naringenin	0.01	0.01	0.01	0.01	0.01	0.01	0.01	0.02	0.02
Flavanone glycoside	Naringin	0.26	1.48	2.54	1.51	0.46	0.52	0.31	0.46	0.35
Flavonol	Kaempferide	NF	NF	0.01	NF	NF	0.01	0.01	NF	NF
Flavonol glycoside	Rutin	0.02	0.02	0.04	0.01	0.39	0.50	0.25	0.02	0.10
	Quercetin 3-*O*-glucoside	0.39	0.47	0.08	0.06	0.08	0.08	0.08	0.14	0.11

**Table 2:** Total phenolic and total flavonoid content; in vitro antioxidant, anti-inflammatory, and tyrosinase inhibitory activities.

Species	Total Phenolic Content (mgGaE/g dw) (C 1 mg/mL)	Total Flavonoid Content (mg QuE/g dw), (C 1 mg/mL)	DPPH Radical Scavenging IC_50_ (mg/mL)	ABTS Radical Scavenging IC_50_ (mg/mL)	*β* Carotene Assay IC_50_ (mg/mL)	FRAP (μmol Fe^2+^/mg dw), (C 1 mg/mL)	BSA% of Inhibition (C 100 µg/mL)	Tyrosinase Inhibitory Activity IC_50_ (mg/mL)
A1	101.06 ± 0.201 ^f^	48.94 ± 0.086 ^h^	0.56 ± 0.181 ^b,c,d^	1.81 ± 0.033 ^g,h^	2.86 ± 0.012 ^c^	289.90 ± 0.012 ^e^	44.44 ± 0.001 ^a^	4.59 ± 0.791 ^b,c^
A2	84.12 ± 0.054 ^c^	36.79 ± 0.019 ^g^	0.16 ± 0.220 ^a,b^	1.63 ± 0.028 ^f,g^	4.18 ± 0.037 ^d^	223.23 ± 0.022 ^b^	58.95 ± 0.003 ^c^	5.54 ± 0.389 ^c,d^
A3	120.51 ± 0.236 ^g^	34.58 ± 0.086 ^f^	0.13 ± 0.135 ^a^	0.54 ± 0.018 ^b^	2.63 ± 0.046 ^b^	741.81 ± 0.034 ^i^	73.42 ± 0.005 ^g^	5.45 ± 0.438 ^c,d^
A4	120.03 ± 0.157 ^g^	23.73 ± 0.076 ^c^	0.15 ± 0.106 ^a^	0.76 ± 0.023 ^c^	4.26 ± 0.059 ^d^	564.19 ± 0.005 ^h^	77.68 ± 0.004 ^h^	5.01 ± 0.018 ^b,c^
A5	68.91 ± 0.265 ^a^	32.31 ± 0.168 ^d^	0.64 ± 0.154 ^c,d^	1.54 ± 0.002 ^e,f^	4.65 ± 0.013 ^c^	354.66 ± 0.010 ^f^	86.23 ± 0.005 ^i^	5.26 ± 0.241 ^c^
A6	100.61 ± 0.235 ^f^	20.48 ± 0.054 ^b^	0.28 ± 0.098 ^a,b,c^	1.07 ± 0.077 ^d^	6.68 ± 0.058 ^g^	418.47 ± 0.006 ^g^	48.80 ± 0.007 ^b^	6.42 ± 0.323 ^d,e^
A7	94.03 ± 0.015 ^e^	5.47 ± 0.084 ^a^	0.74 ± 0.240 ^d^	1.83 ± 0.049 ^h^	8.01 ± 0.011 ^i^	145.61 ± 0.006 ^a^	68.57 ± 0.005 ^d^	4.11 ± 0.241 ^b^
A8	77.08 ± 0.059 ^b^	20.22 ± 0.052 ^b^	1.25 ± 0.115 ^e^	1.21 ± 0.144 ^d^	7.16 ± 0.075 ^h^	265.14 ± 0.005 ^d^	71.27 ± 0.002 ^f^	4.82 ± 0.412 ^b,c^
A9	85.10 ± 0.201 ^d^	33.48 ± 0.152 ^e^	0.68 ± 0.126 ^d^	1.40 ± 0.118 ^e^	5.22 ± 0.005 ^f^	228.00 ± 0.003 ^c^	70.09 ± 0.008 ^e^	6.88 ± 0.127 ^e^
BHA	/	/	0.12 ± 0.055 ^a^	0.09 ± 0.016 ^a^	0.01 ± 0.004 ^a^	797.05 ± 1.00 ^j^	/	/
VitC	/	/	0.06 ± 0.016 ^a^	0.11 ± 0.010 ^a^	/	1348.47 ± 2.00 ^k^	/	/
Diclofenac	/	/	/	/	/	/	95.60 ± 0.001 ^j^	/
Kojic acid	/	/	/	/	/	/	/	0.02 ± 0.001 ^a^

Values are expressed as means ± SD of three independent measurements. GAE, gallic acid equivalents per gram of dry extract; QuE, quercetin hydrate equivalents per gram of dry extract. Each value in the table is expressed as the mean ± SD (n = 3). BHT, butylated hydroxytoluene; DPPH, 1,1-diphenyl-2-picrylhydrazyl; ABTS, 2,2′-azino-bis (3-ethylbenzothiazoline-6-sulfonic acid). SD, standard deviation; IC, inhibition concentration; BSA, bovine serum albumin. Different superscript letters within the same column indicate statistically significant differences among samples (one-way ANOVA followed by Tukey’s post hoc test, *p* < 0.05). Values sharing at least one common letter are not significantly different (*p* ≥ 0.05).

**Table 3:** Antibacterial activity of tested *Ajuga* extracts; results are in mg/mL.

Plant/Bacteria	*B. cereus*		*M. flavus*		*L. monocytogenes*		*S. aureus*		*P. aeruginosa*		*E. coli*		*E. cloacae*		*S. typhimurium*	
	MIC	MBC	MIC	MBC	MIC	MBC	MIC	MBC	MIC	MBC	MIC	MBC	MIC	MBC	MIC	MBC
A1	0.47	0.94	3.75	7.50	7.50	15.00	1.88	15.00	3.75	15.00	7.50	15.00	7.50	15.00	1.88	15.00
A2	0.47	0.94	0.94	1.88	1.88	7.50	1.88	7.50	1.88	7.50	3.75	7.50	3.75	7.50	1.88	7.50
A3	0.47	0.94	1.88	3.75	1.88	3.75	1.88	3.75	3.75	7.50	1.88	7.50	3.75	7.50	1.88	7.50
A4	0.12	0.23	0.94	1.88	1.88	3.75	3.75	7.50	1.88	3.75	0.47	0.94	3.75	7.50	0.94	3.75
A5	0.12	0.23	0.94	1.88	1.88	3.75	0.94	3.75	0.94	3.75	0.23	0.94	3.75	7.50	1.88	15.00
A6	0.23	0.47	1.88	3.75	1.88	7.50	0.94	3.75	0.94	7.50	0.47	0.94	3.75	15.00	0.94	7.50
A7	0.47	0.94	1.88	3.75	1.88	15.00	7.50	15.00	7.50	15.00	0.47	1.88	3.75	15.00	1.88	7.50
A8	0.23	0.47	0.94	1.88	3.75	7.50	0.94	7.50	0.94	7.50	3.75	7.50	3.75	7.50	1.88	7.50
A9	0.94	1.88	0.94	1.88	1.88	15.00	7.50	15.00	7.50	15.00	7.50	15.00	7.50	15.00	3.75	7.50
*Ampicillin*	0.10	0.15	0.10	0.15	0.15	0.30	0.01	0.01	0.01	0.02	0.15	0.30	0.10	0.15	0.10	0.20
*Streptomycin*	0.02	0.05	0.05	0.10	0.15	0.30	0.05	0.10	0.05	0.10	0.05	0.10	0.02	0.05	0.10	0.20

**Table 4:** Antifungal activity of tested *Ajuga* extracts; results are in mg/mL.

Plant/Fungi	*P. funiculosum*		*P. verrucosum var cyclopium*		*P. ochrochloron*		*T. viride*		*A. fumigatus*		*A. versicolor*		*A. ochraceus*		*A. niger*	
	MIC	MFC	MIC	MFC	MIC	MFC	MIC	MFC	MIC	MFC	MIC	MFC	MIC	MFC	MIC	MFC
A1	0.06	0.23	0.94	7.50	0.94	3.75	1.88	3.75	1.88	3.75	0.23	1.88	3.75	>15.00	15.00	>15.00
A2	0.47	1.88	1.88	15.00	1.88	15.00	1.88	3.75	3.75	15.00	0.94	7.50	3.75	>15.00	15.00	>15.00
A3	0.94	3.75	1.88	15.00	1.88	3.75	0.47	7.50	0.94	15.00	3.75	15.00	3.75	>15.00	15.00	>15.00
A4	1.88	3.75	3.75	15.00	3.75	15.00	0.94	7.50	0.94	15.00	1.88	15.00	7.50	>15.00	15.00	>15.00
A5	0.47	3.75	3.75	15.00	3.75	15.00	0.94	7.50	1.88	15.00	3.75	15.00	7.50	>15.00	15.00	>15.00
A6	0.47	0.94	1.88	15.00	3.75	15.00	3.75	15.00	1.88	15.00	1.88	15.00	7.50	>15.00	15.00	>15.00
A7	0.94	7.50	3.75	>15.00	7.50	>15.00	3.75	7.50	0.94	3.75	0.94	7.50	3.75	>15.00	15.00	>15.00
A8	15.00	>15.00	7.50	>15.00	7.50	>15.00	7.50	15.00	3.75	15.00	3.75	>15.00	7.50	>15.00	15.00	>15.00
A9	0.94	3.75	1.88	7.50	3.75	15.00	7.50	15.00	7.50	15.00	7.50	15.00	7.50	>15.00	15.00	>15.00
*Ketoconazole*	0.20	0.40	0.01	0.01	0.01	0.01	0.20	0.40	0.02	0.05	0.01	0.01	0.01	0.03	0.10	0.20
*Nystatin*	0.02	0.05	0.01	0.01	0.01	0.01	0.02	0.05	0.00	0.01	0.05	0.10	0.03	0.05	0.05	0.10

**Table 5:** Pyocyanin production by *Pseudomonas aeruginosa* PAO1 in the presence of *Ajuga* extracts (%, mean ± SD).

*Ajuga* Extracts/Standards	Pyocyanin Production (%)
A1	77.34 ± 0.06 ^g^
A2	90.64 ± 0.23 ^f^
A3	138.57± 0.40 ^c^
A4	147.71 ± 0.28 ^a^
A5	138.52 ±0.51 ^c^
A6	139.97 ± 0.14 ^c^
A7	68.34 ± 0.05 ^h^
A8	99.93 ± 0.31 ^e^
A9	60.20 ± 0.28 ^i^
Ampicillin	143.68 ± 2.99 ^b^
Streptomycin	101.15 ± 0.15 ^e^
PAO1	105.62 ± 0.26 ^d^

Indicated letters mean a significant difference in the same column (*p* < 0.05).

**Table 6:** *Ajuga* species collected from different localities in Serbia and their extraction yields.

	Botanical Name	Locality	Voucher No.	Acronym	Extract Yield (g)
1	*Ajuga laxmannii* (Murray) Benth.	Vidlič Mountain	10851	*A1*	1.58
2	*Ajuga chamaepitys* (L.) Schreb.	Vidlič Mountain	10852	*A2*	1.84
3	*Ajuga reptans* L.	Vidlič Mountain	10853	*A3*	1.01
4	*Ajuga reptans* L.	Suva planina Mountain	10859	*A4*	1.38
5	*Ajuga reptans* L.	Rtanj Mountain	10858	*A5*	1.72
6	*Ajuga reptans* L.	Vlasina Plateau	10855	*A6*	1.12
7	*Ajuga genevensis* L.	Vlasina Plateau	10856	*A7*	1.65
8	*Ajuga genevensis* L.	Suva planina Mountain	10857	*A8*	2.16
9	*Ajuga genevensis* L.	Vidlič Mountain	10854	*A9*	2.3

## Data Availability

The original contributions presented in this study are included in the article. Further inquiries can be directed to the corresponding author.

## Supplementary Materials

The following supporting information can be downloaded at: https://www.mdpi.com/article/10.3390/plants15152306/s1, Figure S1: Principal Component Analysis (PCA) biplot representing the distribution of *Ajuga* samples based on their phenolic profiles; Figure S2: Hierarchical clustered heatmap illustrating the correlation between various bioactivities and *Ajuga* extract samples. The color scale indicates standard Z-scores (normalized activity values); Figure S3: Dose–response curves of the top three performing *Ajuga* extracts against the DPPH radical; Table S1: Pearson correlation coefficients (*r*) between key phenolic compounds and global bioactivities of *Ajuga* extracts.

## Abbreviations

The following abbreviations are used in this manuscript:

Abbreviation	Definition
ABTS	2,2′-Azino-bis(3-ethylbenzothiazoline-6-sulfonic acid)
ANOVA	Analysis of Variance
BHA	Butylated hydroxyanisole
BSA	Bovine serum albumin
DOPA	3,4-Dihydroxy-L-phenylalanine
DPPH	2,2-Diphenyl-1-picrylhydrazyl
DW	Dry weight
FRAP	Ferric Reducing Antioxidant Power
GaE	Gallic acid equivalents
IC_50_	Half-maximal inhibitory concentration
MBC	Minimum bactericidal concentration
MFC	Minimum fungicidal concentration
MIC	Minimum inhibitory concentration
NF	Not found
NSAID	Non-steroidal anti-inflammatory drug
PAO1	*Pseudomonas aeruginosa* PAO1 reference strain
QuE	Quercetin equivalents
ROS	Reactive oxygen species
SD	Standard deviation
TFC	Total flavonoid content
TPC	Total phenolic content
UHPLC-MS/MS	Ultra-high-performance liquid chromatography–tandem mass spectrometry
VitC	Vitamin C (ascorbic acid)
