# Supplementary material for: Comparative Phytochemical Characterization and Bioactivity Profiling of Serbian Ajuga Species from Different Localities: Antioxidant, Antityrosinase, Antimicrobial and Anti-Pyocyanin Activities
Source: Plants (Basel). 2026 Jul 27;15(15):2306. doi: 10.3390/plants15152306 (PMC13468636; doi:10.3390/plants15152306)
Supplement: Supplementary file 1 [file plants-15-02306-s001.zip › plants-4431132-supplementary.pdf]

Supplementary material

Comparative phytochemical characterization and bioactivity profiling of Serbian *Ajuga* species from different localities: antioxidant, antityrosinase, antimicrobial and anti-pyocyanin activities

Ana M. Džamić [1,*], Jelena S. Matejić [2,*], Ksenija S. Mileski [1], Lazar D. Žarković [1], Marija S. Marković [3], Anđela V. Dragićević [2], Uroš Gašić [4], Danijela Mišić [4], and Ana D. Ćirić [4]

[1] University of Belgrade-Faculty of Biology, Institute of Botany and Botanical Garden "Jevremovac", Studentski trg 16, 11000 Belgrade, Serbia
[2] University of Niš, Faculty of Medicine, Bulevar Dr Zorana Đinđića 81, 18000 Niš, Serbia
[3] Institute of Forestry, Kneza Višeslava 3, Belgrade 11030, Serbia
[4] University of Belgrade, Institute for Biological Research "Siniša Stanković", - National Institute of the Republic of Serbia, Bulevar Despota Stefana 142, 11000 Belgrade, Serbia
* These authors contributed equally
* Correspondence: simicana@bio.bg.ac.rs (A.M.Dz) and jekamajic@gmail.com (J.S.M)

Figure S1. Principal Component Analysis (PCA) biplot representing the distribution of *Ajuga* samples based on their phenolic profiles.

Figure S2. Hierarchical clustered heatmap illustrating the correlation between various bioactivities and *Ajuga* extract samples. The color scale indicates standard Z-scores (normalized activity values).

Figure S3. Dose–response curves of the top three performing *Ajuga* extracts against the DPPH radical.

Table S1. Pearson correlation coefficients (r) between key phenolic compounds and global bioactivities of *Ajuga* extracts.

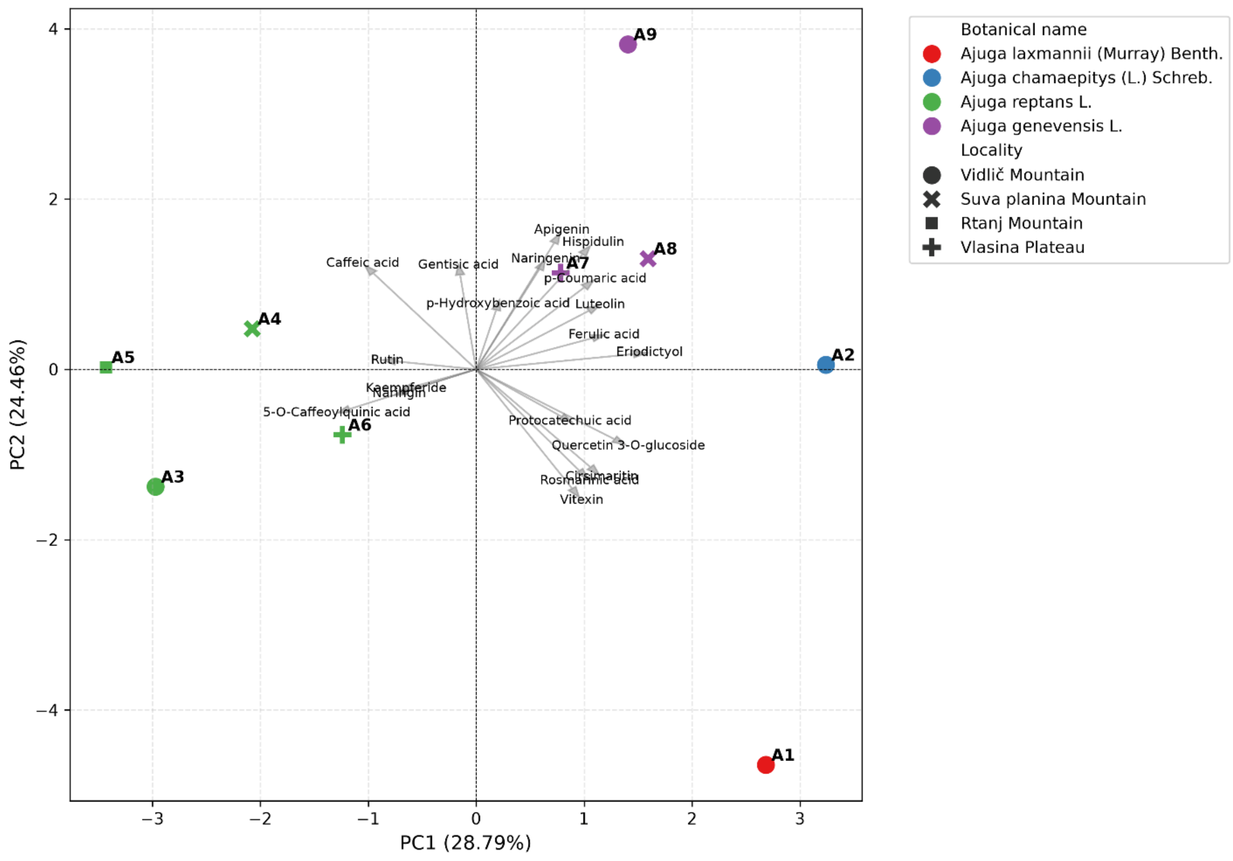

Botanical name
Ajuga laxmannii (Murray) Benth.
Ajuga chamaepitys (L.) Schreb.
Ajuga reptans L.
Ajuga genevensis L.
Locality
Vidlič Mountain
Suva planina Mountain
Rtanj Mountain
Vlasina Plateau
PC1 (28.79%)
PC2 (24.46%)
A1
A2
A3
A4
A5
A6
A7
A8
A9
Apigenin
Hispidulin
Naringenin
Caffeic acid
Gentisic acid
p-Coumaric acid
p-Hydroxybenzoic acid
Luteolin
Ferulic acid
Eriodictyol
Rutin
Kaempferide
5-O-Caffeoylquinic acid
Protocatechuic acid
Quercetin 3-O-glucoside
Cirsimaritin
Vitexin

Figure S1

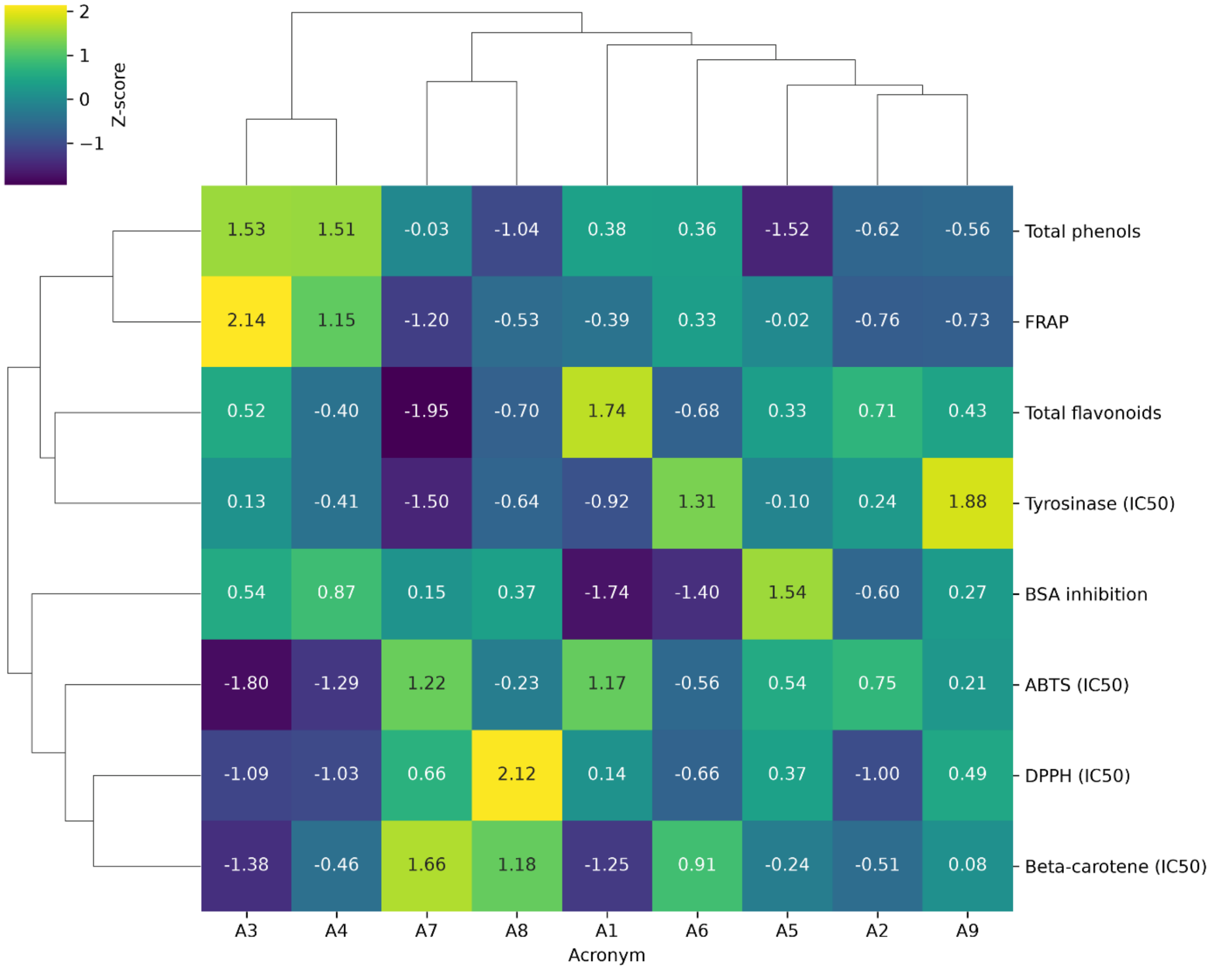

2
1
0
−1
Z-score
Total phenols
FRAP
Total flavonoids
Tyrosinase (IC50)
BSA inhibition
ABTS (IC50)
DPPH (IC50)
Beta-carotene (IC50)
A3
A4
A7
A8
A1
A6
A5
A2
A9
Acronym
1.53 1.51 -0.03 -1.04 0.38 0.36 -1.52 -0.62 -0.56
2.14 1.15 -1.20 -0.53 -0.39 0.33 -0.02 -0.76 -0.73
0.52 -0.40 -1.95 -0.70 1.74 -0.68 0.33 0.71 0.43
0.13 -0.41 -1.50 -0.64 -0.92 1.31 -0.10 0.24 1.88
0.54 0.87 0.15 0.37 -1.74 -1.40 1.54 -0.60 0.27
-1.80 -1.29 1.22 -0.23 1.17 -0.56 0.54 0.75 0.21
-1.09 -1.03 0.66 2.12 0.14 -0.66 0.37 -1.00 0.49
-1.38 -0.46 1.66 1.18 -1.25 0.91 -0.24 -0.51 0.08

Figure S2

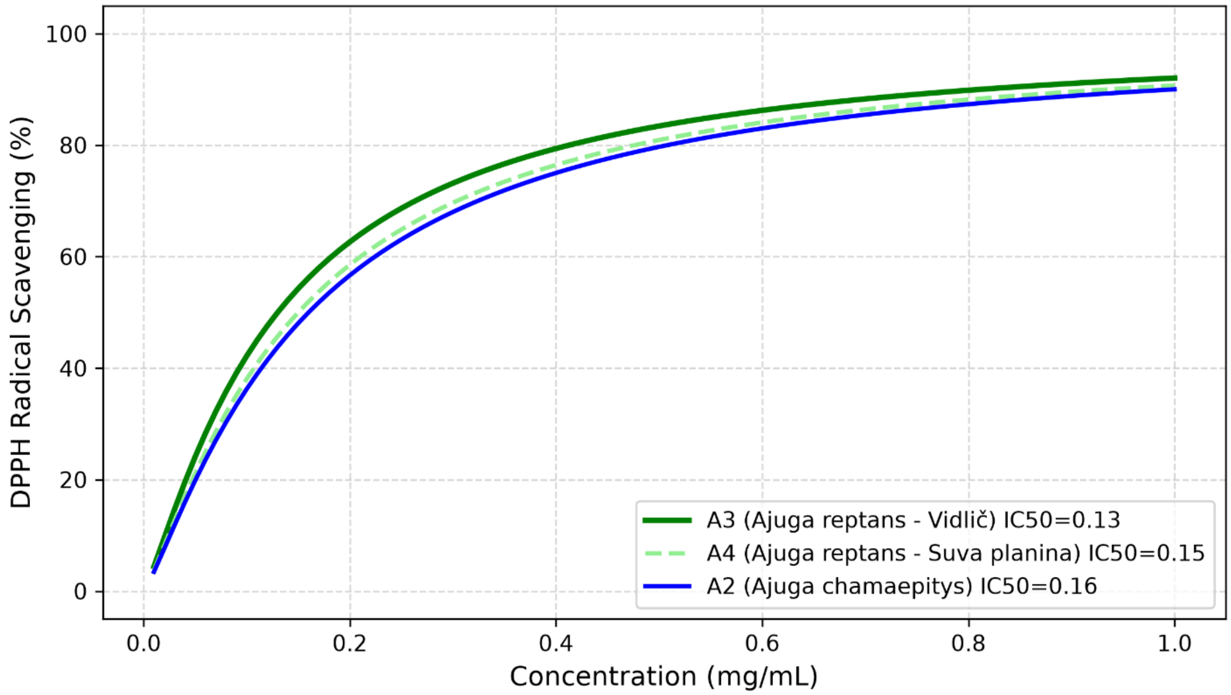
DPPH Radical Scavenging (%)
100
80
60
40
20
0
0.0
0.2
0.4
0.6
0.8
1.0
Concentration (mg/mL)
A3 (Ajuga reptans - Vidlič) IC50=0.13
A4 (Ajuga reptans - Suva planina) IC50=0.15
A2 (Ajuga chamaepitys) IC50=0.16

Figure S3

Table S1

	Antioxidant activity			Antibacterial activity		Antifungal activity		
**Phenolic Compound**	**DPPH (*IC*50)**	**ABTS (*IC*50)**	**FRAP**	***M. flavus* (MIC)**	***E. coli* (MIC)**	***P. funiculosum* (MIC)**	***T. viride* (MIC)**	***P. verrucosum* (MIC)**
**Protocatechuic acid**	**-0.54**	-0.32	0.18	-0.31	-0.42	0.12	-0.22	0.05
**p-Hydroxybenzoic acid**	-0.11	-0.25	0.23	**-0.73**	**-0.68**	0.08	0.15	0.22
**Caffeic acid**	-0.38	**-0.47**	0.31	**-0.71**	**-0.60**	0.25	-0.18	0.34
**Rosmarinic acid**	-0.21	-0.15	0.12	-0.22	-0.15	-0.44	0.11	**-0.63**
**Vitexin**	0.33	-0.21	-0.11	0.45	0.38	**-0.76**	0.29	-0.15
**Rutin**	0.15	-0.11	0.08	-0.44	**-0.65**	-0.28	-0.35	-0.11
**Naringin**	**-0.68**	**-0.72**	**0.78**	-0.25	-0.21	0.31	**-0.74**	0.12

Statistically prominent driving correlations ($r \geq 0.50$) or ($r \leq 0.50$) are highlighted in bold. Negative correlation values for DPPH, ABTS, and all antimicrobial MIC assays indicate that higher compound concentrations directly lead to lower IC_{50} or MIC values, representing stronger biological activity.
